# Age and CMV-Infection Jointly Affect the EBV-Specific CD8^+^ T-Cell Repertoire

**DOI:** 10.3389/fragi.2021.665637

**Published:** 2021-04-29

**Authors:** Josien Lanfermeijer, Peter C. de Greef, Marion Hendriks, Martijn Vos, Josine van Beek, José A. M. Borghans, Debbie van Baarle

**Affiliations:** ^1^Center for Infectious Disease Control, National Institute for Public Health and the Environment, Bilthoven, Netherlands; ^2^Center for Translational Immunology, University Medical Center Utrecht, Utrecht, Netherlands; ^3^Theoretical Biology and Bioinformatics, Utrecht University, Utrecht, Netherlands

**Keywords:** aging, cytomegalovirus, Epstein-Barr virus, T-cell repertoire, T cell

## Abstract

CD8^+^ T cells play an important role in protection against viral infections. With age, changes in the T-cell pool occur, leading to diminished responses against both new and recurring infections in older adults. This is thought to be due to a decrease in both T-cell numbers and T-cell receptor (TCR) diversity. Latent infection with cytomegalovirus (CMV) is assumed to contribute to this age-associated decline of the immune system. The observation that the level of TCR diversity in the total memory T-cell pool stays relatively stable during aging is remarkable in light of the constant input of new antigen-specific memory T cells. What happens with the diversity of the individual antigen-specific T-cell repertoires in the memory pool remains largely unknown. Here we studied the effect of aging on the phenotype and repertoire diversity of CMV-specific and Epstein-Barr virus (EBV)-specific CD8^+^ T cells, as well as the separate effects of aging and CMV-infection on the EBV-specific T-cell repertoire. Antigen-specific T cells against both persistent viruses showed an age-related increase in the expression of markers associated with a more differentiated phenotype, including KLRG-1, an increase in the fraction of terminally differentiated T cells, and a decrease in the diversity of the T-cell repertoire. Not only age, but also CMV infection was associated with a decreased diversity of the EBV-specific T-cell repertoire. This suggests that both CMV infection and age can impact the T-cell repertoire against other antigens.

## Introduction

CD8^+^ T cells play an important role in the control and clearance of viral infections. One of the key components of a protective T-cell response is the recognition of viral epitopes via the T-cell receptor (TCR). T-cell receptors are formed via the random process of somatic V(D)J-recombination, leading to a large collection of TCRs with different specificities (Market and Papavasiliou, 2003). It is generally assumed that the diversity of the T-cell receptor repertoire is positively correlated with the level of protection against infectious diseases (Turner et al., 2009). The diversity of the total CD8^+^ T-cell repertoire decreases with age (Britanova et al., 2014, 2016; Yoshida et al., 2017), which is mainly caused by a decrease in naive T-cell numbers (Britanova et al., 2014) as well as a decreased TCR diversity within the naive T-cell pool (Qi et al., 2014; Egorov et al., 2018). Together with less efficient priming of T cells (Briceno et al., 2016), this may explain why both CD8^+^ T-cell protection against viral infections and vaccine efficacy decrease with age (Goronzy et al., 2001; Deng et al., 2004).

Although the level of TCR diversity within the *total* CD8^+^ T-cell memory pool seems to remain stable with age (Qi et al., 2014), the composition of the memory T-cell pool keeps changing at the antigen-specific level. Exposure to new antigens during life leads to recruitment of new T-cell specificities into the memory pool, and existing memory T-cell clones may expand or contract. The diversity of T cells that are already present in the memory T-cell pool may be affected by the arrival of new memory T-cell specificities, due to competition for T-cell growth and survival factors. The relative stability of the diversity of the *total* CD8^+^ T-cell memory pool therefore does not imply that *individual* antigen-specific T-cell repertoires are stably maintained with age. To study such changes in the memory T-cell pool, we investigated how the diversity of the antigen-specific TCR repertoires against cytomegalovirus (CMV) and Epstein Barr virus (EBV) changes with age.

Both CMV and EBV cannot be cleared from the body and repeatedly challenge the immune system, leading to high, and therefore easily detectable, frequencies of antigen-specific CD8^+^ T cells over all ages in the majority of individuals (Khan et al., 2004; Sukdolak et al., 2013). Previous longitudinal studies focusing on the effect of aging on the antigen-specific repertoire have suggested that the T-cell repertoires against EBV and CMV remain relatively stable, at least during the first few years after primary infection, as the same T-cell clones were identified at different timepoints (Annels et al., 2000; Hadrup et al., 2006; Iancu et al., 2009; Klarenbeek et al., 2012). Consistent with this, several cross-sectional studies into the CMV and EBV-specific T-cell repertoires reported similar Vβ-skewing in young and older adults, and even identical TCR sequences between individuals of different age groups (Khan et al., 2002; Schwanninger et al., 2008; Cardenas Sierra et al., 2014). Although these studies have led to the view that antigen-specific T-cell repertoires are rather stable with age, it remains unknown, if the diversity of antigen-specific T-cell repertoires is maintained (Lanfermeijer et al., 2020).

The cellular immune response against CMV is even more pronounced than the response against EBV, and can reach up to 40% of the CD8^+^ T-cell pool in the blood (Khan et al., 2002; Remmerswaal et al., 2015). Furthermore, CMV-infection leads to changes in the CD8^+^ T-cell pool similar to those observed with aging, including the presence of large fractions of terminally differentiated cells (Almanzar et al., 2005; Chidrawar et al., 2009) and a more skewed and less diverse TCR repertoire (Khan et al., 2002; Nikolich-Zugich, 2008). It has been suggested that the large numbers of CMV-specific T cells can compete with non-CMV-specific T cells (Pawelec et al., 2005; Derhovanessian et al., 2009; Tu and Rao, 2016), leading to memory attrition (Sad and Krishnan, 2003). Studies on the effect of CMV-infection on non-CMV-specific T cells are not unambiguous, however. Several studies have shown that mice infected with murine CMV (MCMV) have impaired responses to heterologous infections (Cicin-Sain et al., 2012; Mekker et al., 2012; Smithey et al., 2012; Redeker et al., 2017). In contrast, another study observed a positive effect of MCMV infection on the diversity of the T-cell repertoire specific for a heterologous infection (Smithey et al., 2018). Studies in humans showed similarly contradicting results: while one study showed that CMV^+^ individuals had lower absolute numbers of EBV-specific T cells than CMV^−^ individuals (Khan et al., 2004), another study found that the diversity of the non-CMV-specific memory T-cell repertoire was comparable in CMV^−^ and CMV^+^ individuals, thereby suggesting that the memory T-cell pool simply expands to accommodate the large frequencies of CMV-specific T cells (Lindau et al., 2019). Thus, the effect of CMV infection on non-CMV-specific T-cell responses and their repertoire diversity, and how this is linked to aging, remains poorly understood (Lanfermeijer et al., 2020).

To gain insight into the maintenance of the repertoire of antigen-specific T cells, we studied the effect of aging on the phenotype and TCR repertoire composition of T cells specific for two immune-dominant CMV and EBV epitopes. In addition, we investigated how the EBV-specific T-cell repertoire is influenced by CMV-infection and how this is linked to aging. We observed that the richness of the CMV-specific and EBV-specific T-cell repertoire declines with age, independent of CMV serostatus. CMV infection led to a further decrease in diversity of the EBV-specific T-cell repertoire. This suggests that CMV infection and age both play an important role in the diversity of the antigen-specific T-cell repertoires.

## Methods and Materials

### Study Design

Samples of healthy individuals covering a broad age range were combined from two cohorts. Samples of young adults (*n* = 34) between 18 and 52 years of age, from a cohort of unvaccinated controls or pre-vaccination participants, were used from a study carried out in 2009–2011 (the Pandemic influenza vaccination trial, Netherlands Trial Register NL1952) (Rosendahl Huber et al., 2018). The study was approved by the Central Committee on Research Involving Human Subjects of the Netherlands. Samples of older adults (*n* = 57), ≥60 years of age, were control samples from a study carried out in 2014–2015 (Influenza-like-illness-3, NL4666) (Kaaijk et al., submitted). This study was approved by the acknowledged ethical committee, METC Noord Holland. Both studies were carried out in accordance with the recommendations of Good Clinical Practice with written informed consent from all subjects, in accordance with the Declaration of Helsinki. See Supplementary Figure 1 for a flowchart of the selection criteria of the donors used for the analysis.

### Cytomegalovirus (CMV)-Specific and Epstein Barr Virus (EBV)-Specific Antibodies

For healthy young adults, CMV-specific antibody levels were measured using a commercial ELISA kit (IBL international GMBH) according to manufacturer's instructions. Participants with a CMV antibody level of ≥12 U/ml or higher were considered CMV^+^, those with a level of ≤8 U/ml were considered CMV^−^. None of the participants included in this study scored between the 8 and 12 U/ml range. For older healthy adults, CMV-specific antibody levels and EBV-specific antibody levels were simultaneously measured in serum by an in-house-developed multiplex immunoassay (Tcherniaeva et al., 2018). Individuals with a CMV-specific antibody level of ≤4 RU (relative units)/ml were considered to be CMV^−^ and individuals with an antibody level >7.5 RU/ml were considered CMV^+^. None of the participants included in this study had a CMV-specific antibody level between 4 and 7.5 RU/ml. Individuals were considered EBV- with an antibody level of ≤16 RU/ml, whereas those with an antibody level of >30 RU/ml were considered EBV^+^. None of the older participants included in this study had an EBV−specific antibody level between 16 and 30 RU/ml. Note that the EBV-status of the younger individuals remained unknown, therefore only individuals with high EBV^A2−GLC^-specific T-cell frequencies were used in our analysis.

### PBMC and Serum Isolation

Peripheral blood mononuclear cells were obtained by Lymphoprep (Progen) density gradient centrifugation from heparinized blood, according to the manufacturer's instructions. PBMCs were frozen in 90% fetal calf serum and 10% dimethyl sulfoxide at −135°C until further use. Serum was isolated out of tubes with clot-activation factor and stored at −80°C until further use.

### Antigen-Specific T Cells by Flow Cytometry

HLA-A2 positive healthy individuals were selected for subsequent EBV-specific and CMV-specific T cell analysis, by staining PBMCs for expression of HLA-A2 with the HLA-A2(BB7.2)-V450 antibody (BD Bioscience). From the HLA-A2 positive individuals, ±4 million PBMCs were stained using the HLA-class I dextramer containing the GLCTLVAML epitope of the BMLF1 protein of EBV (A^*^0201/GLCTLVAML-APC, Immudex) or the NLVPMVATV epitope of the pp65 protein of CMV (A^*^0201/NLVPMVATV-APC, Immudex), for 20 min at room temperature to assess their virus-specific T-cell frequencies.

Surface staining was performed for 30 min at 4°C with the following antibodies: Fixable Viability Staining-780 (BD bioscience), CD3 (SK7)-AF700(BD bioscience), CD8(RPA-T8)-BrilliantViolet510, CD45RO(UCHL1)-BrilliantViolet711, CD27(O323)-BrilliantViolet786, CCR7(150503)-BrilliantUV395 (BD bioscience), KLRG-1(13F12F2)-PE-Cy7 (eBioscience), PD-1(EH12.2H7)-PerCP Cy5.5, CD95(DX2)-BrilliantViolet421 (BD Biosciences), CD127(A019D5)-BrilliantViolet650, CD57(HCD57)-PE, and CXCR3(G025H7)-PE-Dazzle. All antibodies were purchased from Biolegend, unless stated otherwise. Acquisition was performed on a LSRFortessaX20 and data analysis was performed using FlowJo (Treestar). tSNE-analyses were performed using Cytobank (www.cytobank.org) (Kotecha et al., 2010) on 30 randomly selected dextramer-positive CD8^+^ T cells per sample and labeled with epitope-specificity, age, and CMV-serostatus. The tSNE clustering was performed on all these data combined (including both antigen-specificities). Perplexity of the clustering was set at 100. Cofactors for ArcSinH transformation were calculated using the flowVS package for R (https://www.bioconductor.org/packages/release/bioc/html/flowVS.html). Both packages were slightly adapted to allow for FlowCytometric data analysis and integrated in an in-house developed pipeline.

### Isolation of Antigen-Specific T Cells for T-Cell Receptor Analysis

CD8^+^ T cells were isolated from PBMCs using a negative selection microbeads kit (Miltenyi Biotec) according to the manufacturer's protocol. Next, CD8^+^ T cells were labeled at room temperature for 20 min with the A^*^0201/GLCTLVAML-APC dextramer and with the A^*^0201/NLVPMVATV-APC dextramer for CMV^+^ individuals. Subsequently surface staining was performed using the following mAbs: CD3(UCHT1)-PerCP (Biolegend), CD4(OKT4)-BV510 (Biolegend), and CD8(RPA-T8)-FITC (Biolegend). CD3^+^CD4^−^CD8^+^dextramer^+^ cells were then sorted by FACS Melody (BD) directly into RNAlater (Ambion Inc. Applied Biosystems) and stored at −80°C for subsequent TCRβ clonotype analysis.

### Preparing TCRβ cDNA Libraries for Sequencing

TCRβ analysis was performed as described previously (Shugay et al., 2014), with minor modifications. Briefly, mRNA was isolated with the RNA microkit (Qiagen) according to the manufacturer's protocol. Isolated mRNA was used for cDNA synthesis with 5′RACE template switch technology to introduce a universal primer binding site, and unique molecular identifiers (UMIs) were added at the 5′ end of the cDNA molecules using the SMARTScribe Reverse Transcriptase (TaKaRa). cDNA synthesis was followed by an AMPure XP bead-based clean-up (Beckman Coulter). Purified cDNA molecules were amplified in two subsequent PCR steps (25 cycles in PCR1 and 20 cycles in PCR2) using the Q5® High-Fidelity DNA Polymerase (New England BioLabs), with an AMPure XP bead-based clean-up in between. PCR products were size-selected on gel and purified using the Nucleospin PCR clean-up kit (Machery-Nagel). The PCR products were sequenced via Illumina MiSeq paired end 2x250 nucleotide (nt) sequencing.

### TCRβ Clonotype Analysis

Raw sequencing data were processed using the 12nt UMIs to correct for amplification biases and error-correction of reads. RTCR (Gerritsen et al., 2016) was used to identify both the UMI sequence and clonotype information from the reads. Because of the relatively small number of cells per sample, additional filtering steps were followed to minimize cross-sample contamination and biases introduced by errors in the UMI sequence. Sequences were only accepted if their UMI was observed in at least 40 sequencing reads. Sequences with identical UMIs in multiple samples were removed if they did not occur in at least 1,000 sequencing reads or if their absolute frequency was lower than 10% of the maximum frequency in the other samples. UMIs were clustered within each sample if they were within a Hamming distance of 3. More detailed information about the processing and filtering of reads is provided in the Supplementary Material.

Clonotypes were defined by their CDR3 amino acid sequence and V and J segment. Our sequencing reads do not always allow to distinguish between very similar V-segments, e.g., TRBV12-3 and TRBV12-4, which are annotated as V12-3/4 in **Figure 3**, Supplementary Figures 4, 5, and Supplementary Tables 1, 2A,B.

For measuring diversity, the richness (defined as number of distinct clonotypes) of each sample was determined using normalized sample sizes (i.e., by iteratively sampling, without replacement, a given number of UMIs from the full set of UMIs that were identified in the sample). This approach accounts for the fact that the number of RNA molecules sampled may differ between cells. Diversity was calculated using the previously described Simpson's diversity index (Venturi et al., 2007). This index ranges between 0 and 1, with 0 representing minimal diversity and 1 representing maximal diversity. Sequence generation probabilities (CDR3+ V and J segments) were calculated using the default recombination model of OLGA (Sethna et al., 2019). Known antigen specificity of sequences was assessed using the VDJdb (Shugay et al., 2017; retrieved on 29 October 2020). Sequences from CMV^A2−NLV^-and EBV^A2−GLC^-specific samples were counted as a match if their V gene + CDR3 amino acid sequence + J gene was listed as a human TCRbeta sequence specific for the NLVPMVATV or GLCTLVAML epitope, respectively.

### Statistical Analysis of Flow Cytometry Data

Differences between the groups (for example CMV^−^ compared to CMV^+^) were assessed using Mann-Whitney *U*-tests. Correlations were tested with Spearman's rank correlation coefficient. For all analyses, *p*-values < 0.05 were considered statistically significant. Data were analyzed using GraphPad Prism 8.3 and SPSS statistics 22 for Windows (SPSS Inc., Chicago, IL, USA).

## Results

### Characteristics of the Study Population

Healthy HLA-A2 positive individuals were on average 57.8 ± 19.0 years old (*n* = 91, range 21–82 years) and 57.1% of these individuals were CMV^+^. Samples were obtained from two different cohorts, one containing young adults (21–52 years old) (*n* = 34), and one containing older adults (≥60 years old) (*n* = 57), of whom respectively 55.9 and 57.9% were CMV^+^ (Table 1). No significant differences in age or sex were observed between CMV^−^ and CMV^+^ individuals. Supplementary Figure 1 gives a flowchart of the selection criteria of the donors used for the analysis.

**Table 1:** Characteristics of the study population.

**Healthy young adults**	**Total (*n* = 34)**	**CMV^**−**^ (*n* = 15)**	**CMV^**+**^ (*n* = 19)**	**Statistics**
Age (mean ± SD)	35.9 ± 10.3	35.3 ± 10.8	36.4 ± 10.1	ns
Sex (% women)	61.8%	53.3%	68.4%	ns
CMV-serostatus (CMV^+^)	55.9%	.	.	.
**Healthy older adults**	**Total (*****n*** **= 57)**	**CMV**^**−**^ **(*****n*** **= 24)**	**CMV**^**+**^ **(*****n*** **= 33)**	**Statistics**
Age (mean ± SD)	71.2 ± 6.4	70.8 ± 6.6	71.5 ± 6.4	ns
Sex (% women)	43.9%	37.5%	48.5%	ns
CMV-serostatus (CMV^+^)	57.9%	.	.	.

*Overview of the sex and CMV-serostatus distribution of the young adults (Rosendahl Huber et al., 2018) and older individuals (Kaaijk et al., submitted). Differences in percentage of CMV serostatus between groups was tested with Chi-squared test (p = ns for all age groups). Differences in age between groups was tested with unpaired T-test. ·means not applicable*.

### Changes in the Phenotype of Antigen-Specific CD8^+^ T Cells With Age

**Figure 1:**
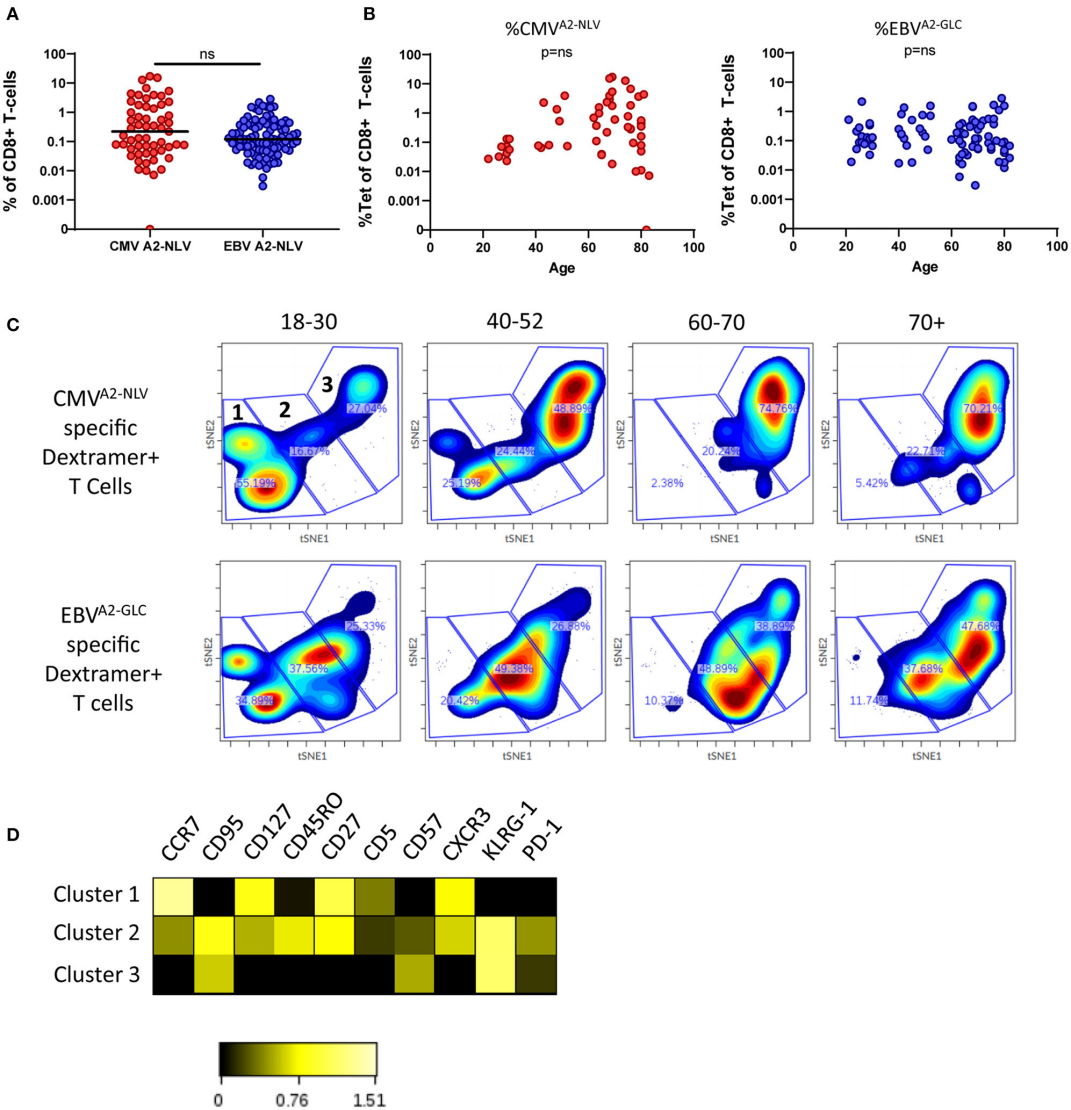
Changes in the phenotype of antigen-specific CD8^+^ T cells with age. **(A)** Percentage of CMV^A2−NLV^-specific CD8^+^ T cells (red, *n* = 56) and EBV^A2−GLC^-specific CD8^+^ T cells (blue, *n* = 99). Horizontal lines represent group median. **(B)** Percentage of CMV^A2−NLV^ (left) and EBV^A2−GLC^-specific (right) CD8^+^ T cells as a function of age. **(C)** t-SNE analysis of CMV^A2−NLV^-specificand EBV^A2−GLC^-specific CD8^+^ T cells of donors (*n* = 47 and *n* = 77, respectively) categorized in four age groups. Clustering is based on MFI of CD5, PD-1, CD57, KLRG-1, CXCR3, CCR7, CD45RO, CD95, CD27, and CD127 of both epitopes. From each sample 30 cells were used. Three large clusters were manually identified. **(D)** Heatmap of expression of markers of the three t-SNE clusters. Clustering of t-SNE based on both CMV^A2−NLV^ and EBV^A2−GLC^-specific CD8^+^ T cells. Heatmap was based on the Log10 ratio of the median expression of the markers, normalized per marker to its column's minimum. Difference between epitopes was compared by Mann Whitney *U*-test. Correlations were tested with Spearman's rank correlation coefficient, ns stands for a non-significant *p*-value.

To study the association between age and the antigen-specific CD8^+^ T-cell frequencies against EBV and CMV, we performed a dextramer-staining with one immuno-dominant epitope of CMV (NLVPMVATV) derived from the pp65 protein and one immune-dominant epitope of EBV (GLCTLVAML) derived from the BMLF-1 protein, both presented on the HLA-A2 molecule. We investigated the T-cell frequency against these two epitopes at different ages. The frequencies of CMV^A2−NLV^-specific T cells tended to be slightly higher than the frequencies of EBV^A2−GLC^-specific T cells [median of 0.220% vs. median of 0.120%; *P* = 0.0990 (ns)] (Figure 1A). We observed less inter-individual heterogeneity in the CMV^A2−NLV^-specific T-cell frequencies of younger compared to older adults; in the older adults, in whom these frequencies ranged from very low (from 0.01%) to very high (up to 17%) (Figure 1B, left panel). EBV^A2−GLC^-specific T-cell frequencies were not significantly associated with the age of the individuals (Figure 1B, right panel).

To assess the association between age and the phenotype of CMV^A2−NLV^ and EBV^A2−GLC^-specific T cells, we performed a cluster analysis (tSNE) based on the expression of the memory T-cell markers CD27, CCR7, CD95, CD45RO, and CXCR3, on CD57 and KLRG-1, which are associated with a more differentiated phenotype and on the inhibitory receptor PD-1, and CD5, which plays a role in TCR signaling (Voisinne et al., 2018). For the tSNE analysis the same amount of antigen-specific T cells (i.e., 30) per sample was used. The very same clustering was applied on the samples in the four age groups and per epitope-specificity. We observed clear differences in the subset distribution between these groups for both CMV^A2−NLV^ and EBV^A2−GLC^-specific T cells (Figure 1C). We identified 3 large clusters (1–3, Figure 1C, upper left panel), in which cluster 1 contains Central memory type markers CCR7^high^, CD27^high^, KLRG-1^low^, CD57^low^ cells, while cluster 3 contains the more differentiated cells, expressing CCR7^low^, CD27^low^, and KLRG-1^high^. Cluster 2 forms an intermediate cluster based on the expression of these markers (Figure 1D). Despite relatively large inter-individual variation (Supplementary Figure 2A), our data suggest a shift from cluster 1 to cluster 3 for both CMV^A2−NLV^ and EBV^A2−GLC^-specific T cells with age (Figure 1C, Supplementary Figure 2A). This shift in clusters occurs earlier and becomes more pronounced with age for CMV^A2−NLV^-specific T cells than for EBV^A2−GLC^-specific T cells.

### Phenotypic Changes of CMV-Specific and EBV-Specific T Cells Are Differently Associated With Age

**Figure 2:**
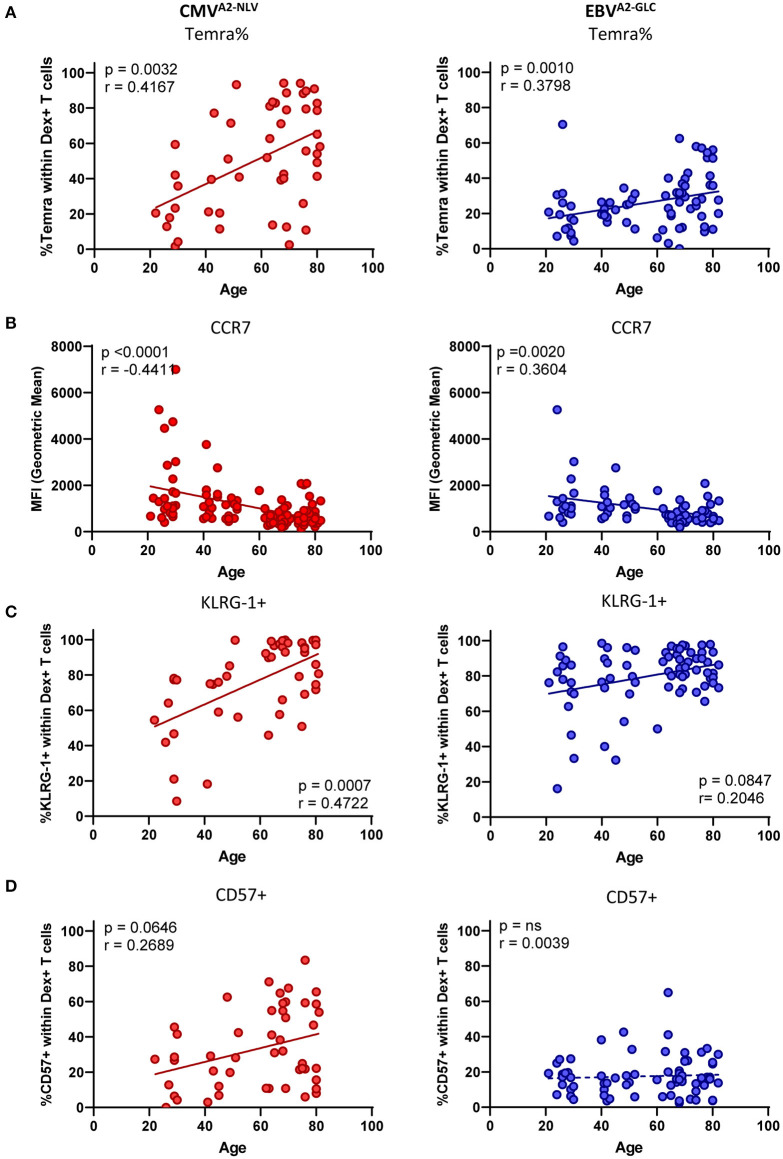
The phenotype of CMV-specific and EBV-specific T cells is affected differently by age. **(A–D)** Phenotype analysis of both CMV^A2−NLV^-specific (red) and EBV^A2−GLC^-specific CD8^+^ T cells (blue) plotted against age. **(A)** Percentage of Temra cells (CD27–, CD45RO–). **(B)** Geometric Mean of the fluorescent intensity of CCR7. Percentage of KLRG-1^+^ **(C)** and CD57^+^ **(D)** within Dextramer^+^ CD8^+^ T cells. Solid lines indicate a slope significantly (*p* < 0.05) different from a slope of 0, whereas a dotted line indicates no significant difference. Only donors with a sufficient T-cell response (at least 25 cells) were used for the phenotypical staining. Correlations were tested with Spearman's rank correlation coefficient.

We next explored how these phenotypic changes associated with age by quantifying the expression of various markers in the individual samples of CMV^A2−NLV^-specific and EBV^A2−GLC^-specific T cells. This allowed us to use the expression data of all the dextramer^+^ T cells. The composition of the memory population for the different ages based on conventional gating supported our observations of the cluster analysis. The memory subsets were defined based on the expression of CD27 and CD45RO in which the CD27^−^CD45RO^−^ subset is referred to as Temra cells. We found a positive association between age and the percentage of Temra cells for both CMV^A2−NLV^-specific T cells [*p* = 0.0032, *r* = 0.4167, slope of 0.75%/year (*p* = 0.0006)] and, albeit to a lesser extent, EBV^A2−GLC^-specific T cells [*p* = 0.0010, *r* = 0.0.3796, slope of 0.25%/year (*p* = 0.1045)] (Figure 2A). The fraction of Temra cells correlated positively with the frequency of CMV^A2−NLV^-specific T cells, but not with the percentage of EBV^A2−GLC^-specific T cells (Supplementary Figure 3A). For EBV^A2−GLC^-specific T cells, the proportion of effector memory cells (Tem, CD27^−^CD45RO^+^) increased significantly with age (*p* < 0.0001, *r* = 0.5001) (Supplementary Figure 3B, right panel). The cluster analysis based on MFI from Figure 1 also showed a gradual decrease in CCR7 expression with age, which was confirmed by plotting the geometric mean of the fluorescence intensity (gMFI) of CCR7 on a continuous scale for both CMV^A2−NLV^-specific (*p* < 0.0001, *r* = 0.0.4411) and EBV^A2−GLC^-specific T cells (*p* = 0.0020, *r* = 0.3604) (Figure 2B).

Next, we analyzed the expression of markers associated with a more differentiated phenotype and exhaustion-associated markers on the antigen-specific T cells more closely. Both CMV^A2−NLV^- and EBV^A2−GLC^-specific T cells are associated with high expression of the inhibitory marker KLRG-1. CD57 is a senescence marker known to be specifically highly expressed by CMV-specific T cells, compared to T cells against other chronic viruses (Hoji et al., 2007; van den Berg et al., 2019). We indeed found high percentages of KLRG-1^+^ cells for both CMV^A2−NLV^-specific (mean of 76.6%) and EBV^A2−GLC^-specific T cells (mean of 79.8%), and the percentage of KLRG-1^+^ cells was positively associated with age, both CMV^A2−NLV^-specific (*p* = 0.0007, *r* = 0.4722) and EBV^A2−GLC^-specific T cells (*p* = 0.0847, *r* = 0.2046) (Figure 2C). The percentage of CD57^+^ T cells showed a positive trend with age for CMV^A2−NLV^-specific T cells (*p* = 0.0645, *r* = 0.2689), while it was low across all ages for EBV^A2−GLC^- specific T cells (Figure 2D). We observed a significant positive association between the frequency of CMV^A2−NLV^-specific T cells and the percentage of KLRG-1^+^, CD57^+^ cells in the CMV^A2−NLV^-specific T-cell population (Supplementary Figure 3A, upper panels). The frequency of EBV^A2−GLC^-specific T cells, in contrast, was only positively associated with the percentage of KLRG-1^+^ EBV^A2−GLC^-specific T cells (Supplementary Figure 3A, lower panels), and not with the percentage of CD57^+^ EBV^A2−GLC^-specific T cells.

We also investigated the percentage of CMV^A2−NLV^-specific and EBV^A2−GLC^-specific T cells expressing PD-1, which in the context of chronic (active) infection is often used as an exhaustion marker (Jubel et al., 2020). We found no significant correlation between the percentage of PD-1^+^ cells in the antigen-specific T-cell pool and age, both for EBV-specific and for CMV-specific T cells (Supplementary Figure 3C).

### The CMV^A2-NLV^-Specific Repertoire Is Less Diverse Than the EBV^A2-GLC^-Specific Repertoire

**Figure 3:**
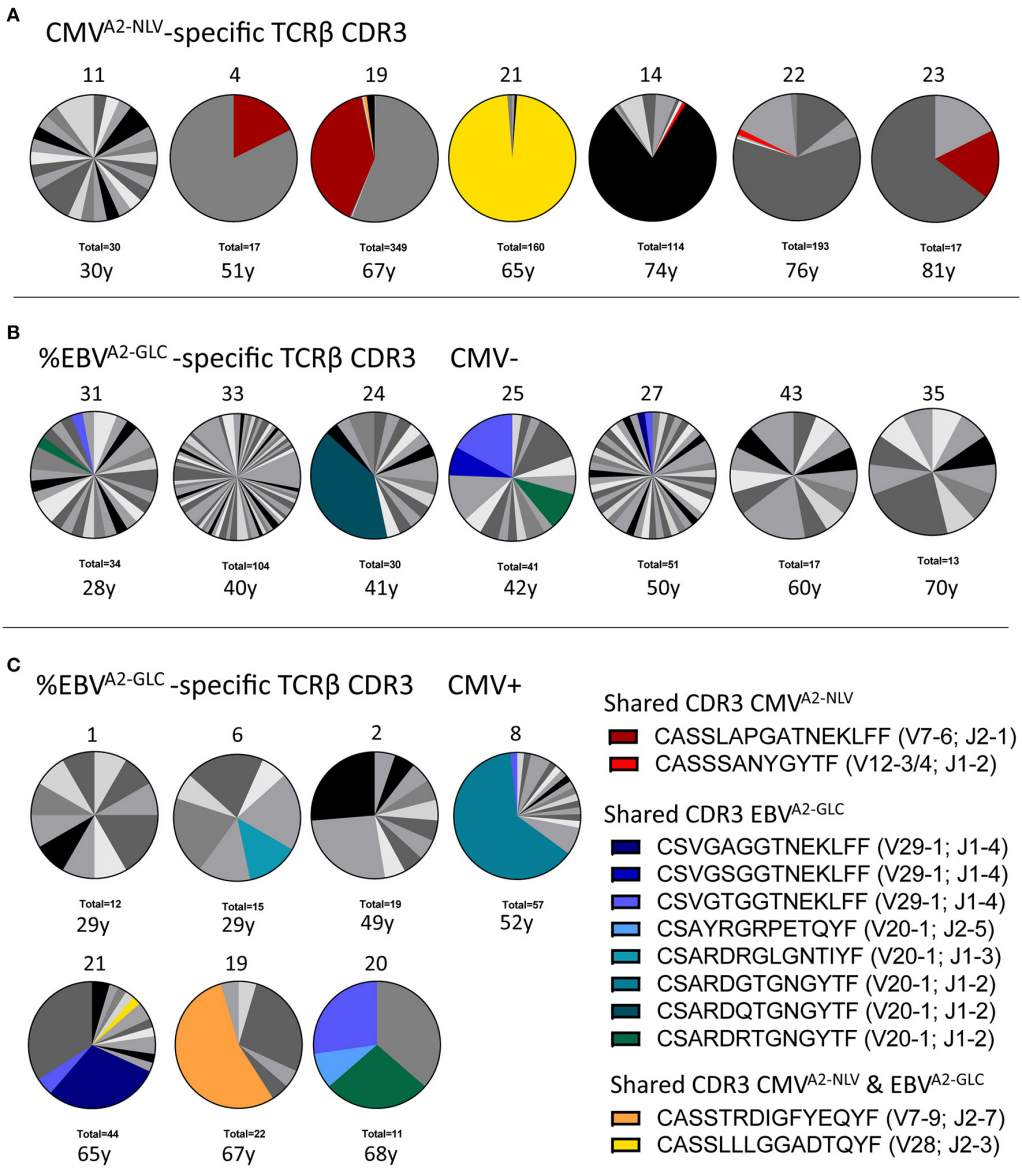
Characterization of the antigen-specific TCRβ repertoire. **(A)** Distribution of TCRβ sequences in samples of CMV^A2−NLV^-specific CD8^+^ T cells (*n* = 7). **(B,C)** T-cell repertoire of EBV^A2−GLC^-specific CD8^+^ T cells of both CMV^−^ **(B)** and CMV^+^ individuals **(C)**. Each pie depicts the repertoire of a different sample, with its Donor ID on top and the total number of UMI-TCR pairs identified at the bottom, as well as the individual's age. Colors represent shared TCRβ sequences between donors. Gray scales depict unique TCRβ sequences. Note two shared sequences between the CMV^A2−NLV^ and EBV^A2−GLC^ sample of two single individuals (yellow and orange). As this sharing was limited to these single donors and involved a very abundant TCRβ in either one of the samples, we expect that this overlap occurred during the sorting of the cells, probably due to unspecific binding of the dextramer.

We then investigated the TCR repertoire of CMV^A2−NLV^-specific and EBV^A2−GLC^-specific T cells in our samples by sequencing the TCRβ-chain. We used unique molecular identifiers (UMIs) to correct for sequencing errors and unequal PCR amplification, and performed an additional filtering procedure to exclude sequences that were likely due to contamination between samples or mutation in the UMI sequence (see Supplementary Material for more details). We proceeded with the samples in which at least 10 UMI-TCR pairs remained after filtering. The distribution of the TCRβ sequences per individual are shown in Figure 3 (see Supplementary Figure 4 for samples with <10 UMI-TCR pairs, all identified TCR sequences are provided in Supplementary Tables 1, 2A,B), with colors indicating the TCRβ sequences that are shared between individuals. We observed two different TCR sequences that were shared between individuals in the CMV^A2−NLV^-specific repertoire samples, and eight in the EBV^A2−GLC^-specific repertoire samples (Supplementary Figures 5A,B). A substantial fraction of the observed TCR sequences were also present in the VDJ database (VDJdb) of reported antigen-specific TCR sequences (Shugay et al., 2017) (Supplementary Figure 5C). We found that the sequences that were shared between individuals within our study or between an individual of our study and the VDJdb had an over 16-fold higher generation probability than those that were not shared (Supplementary Figure 5D). This supports the idea that the likelihood of TCR generation plays an important role in the presence, abundance and sharing of antigen-specific TCR sequences (Venturi et al., 2006, 2008; Elhanati et al., 2018).

**Figure 4:**
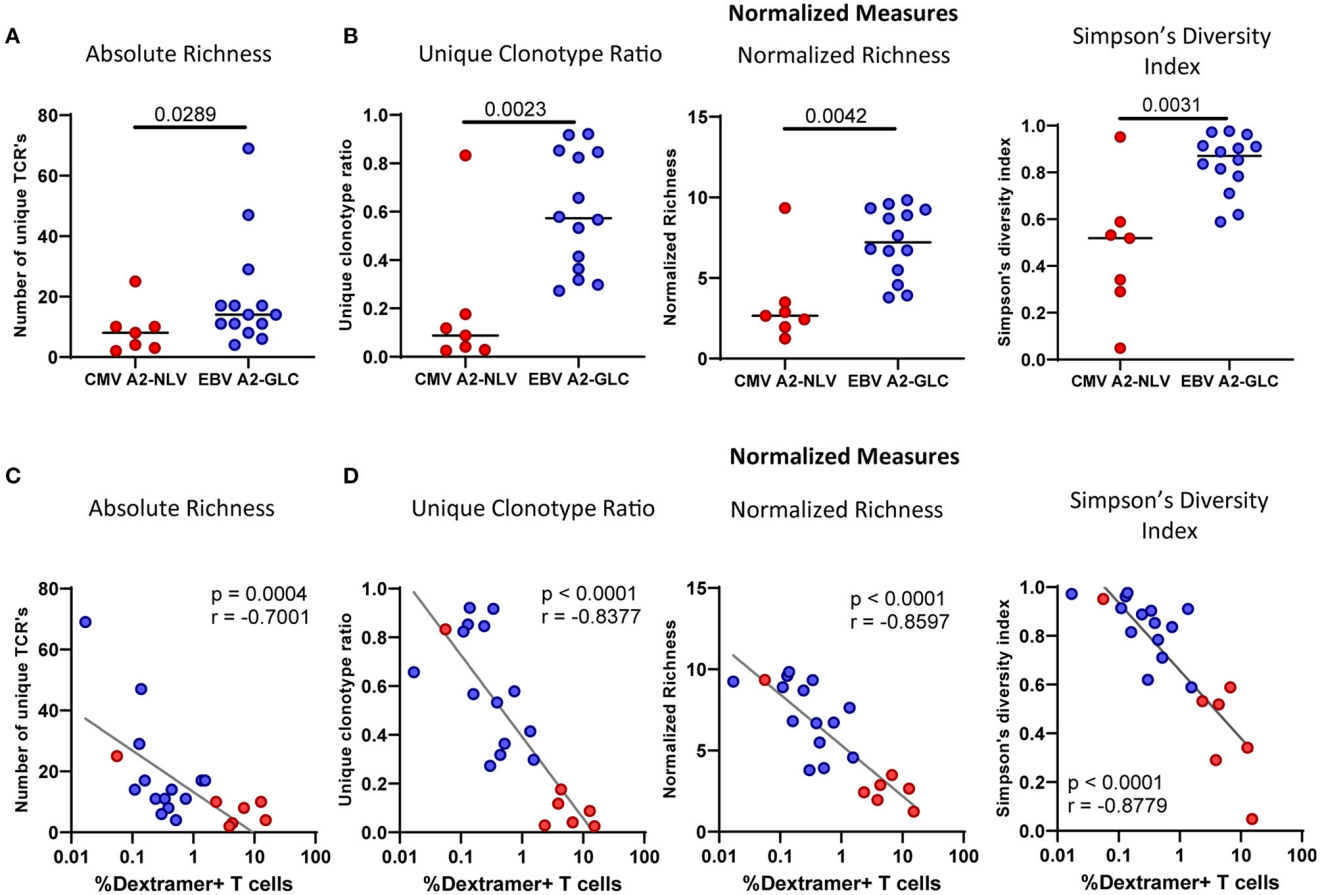
The CMV^A2−NLV^-specific TCRβ repertoire is less diverse than the and EBV^A2−GLC^-specific TCRβ repertoire. Repertoire diversity calculations of the CMV^A2−NLV^ and EBV^A2−GLC^-specific CD8^+^ T-cell repertoire. **(A)** Richness calculations based on the number of different TCRβ sequences. **(B)** Measures normalized for differential sample sizes: Unique Clonotype Ratio (left), normalized richness (middle), and Simpson's Diversity Index (right panel). **(C,D)** Correlation plot of the repertoire richness **(C)** and normalized diversity **(D)** and the frequency of the CMV^A2−NLV^-specific (red) and EBV^A2−GLC^-specific (blue) T cells. Horizontal lines in **(A,B)** show group medians. The unique clonotype ratio was calculated by dividing the number of unique clonotypes by the total number of TCR sequences. Normalized richness was calculated by counting the number of distinct TCR sequences in subsamples of 10 sequences. The mean of 1,000 subsampling iterations is shown. Differences between epitopes were compared by Mann Whitney. Correlations were tested with Spearman's rank correlation coefficient.

To investigate the diversity of the antigen-specific T-cell repertoire, we used several measures of TCR diversity. The absolute richness, i.e., the total number of distinct clonotypes observed in a sample, was significantly higher for the EBV^A2−GLC^-specific T cells than for the CMV^A2−NLV^-specific T cells (*p* = 0.0289) (Figure 4A), even though the frequency of EBV^A2−GLC^-specific T cells was lower than that of CMV^A2−NLV^-specific T cells. However, the absolute richness is largely influenced by the total number of UMI-TCR pairs in a sample, i.e., the number of cDNA molecules that were sequenced. To overcome this potential bias, we therefore also used several alternative measures of TCR diversity. First, we calculated the unique clonotype ratio, by dividing the number of unique clonotypes by the total number of TCR sequences in each sample. Next, we calculated a normalized richness by counting the number of distinct TCR sequences in equally sized subsamples of the actual samples (by taking the mean richness of 10 randomly chosen subsamples). We also calculated the Simpson's diversity index as a sample size-independent measure of the TCR diversity in each sample (Venturi et al., 2007). While richness quantifies the variety of different TCRs, Simpson's diversity index quantifies the evenness of the frequency distribution across the TCRs. Even after excluding differential sample sizes as a confounding factor, we found a higher TCR diversity in the EBV^A2−GLC^-specific compared to the CMV^A2−NLV^-specific T-cell repertoire (Figure 4B). We repeated these analyses on the subset of samples that contained at least 20 UMI-TCR pairs, which did not change the results qualitatively.

To understand what explains the higher diversity in some of the samples, we investigated the correlation between T-cell frequencies and the diversity of the repertoire for the CMV^A2−NLV^-specific and EBV^A2−GLC^-specific T-cell data combined. We found a negative correlation between all diversity measures and the frequency of antigen-specific T cells (Figures 4C,D). This suggests that individuals with high frequencies of antigen-specific T cells had large clonal expansions, leading to a decrease in TCR repertoire diversity.

### Both Age and CMV-Infection Are Associated With a Lower Diversity of the EBV^A2-GLC^-Specific T-Cell Repertoire

**Figure 5:**
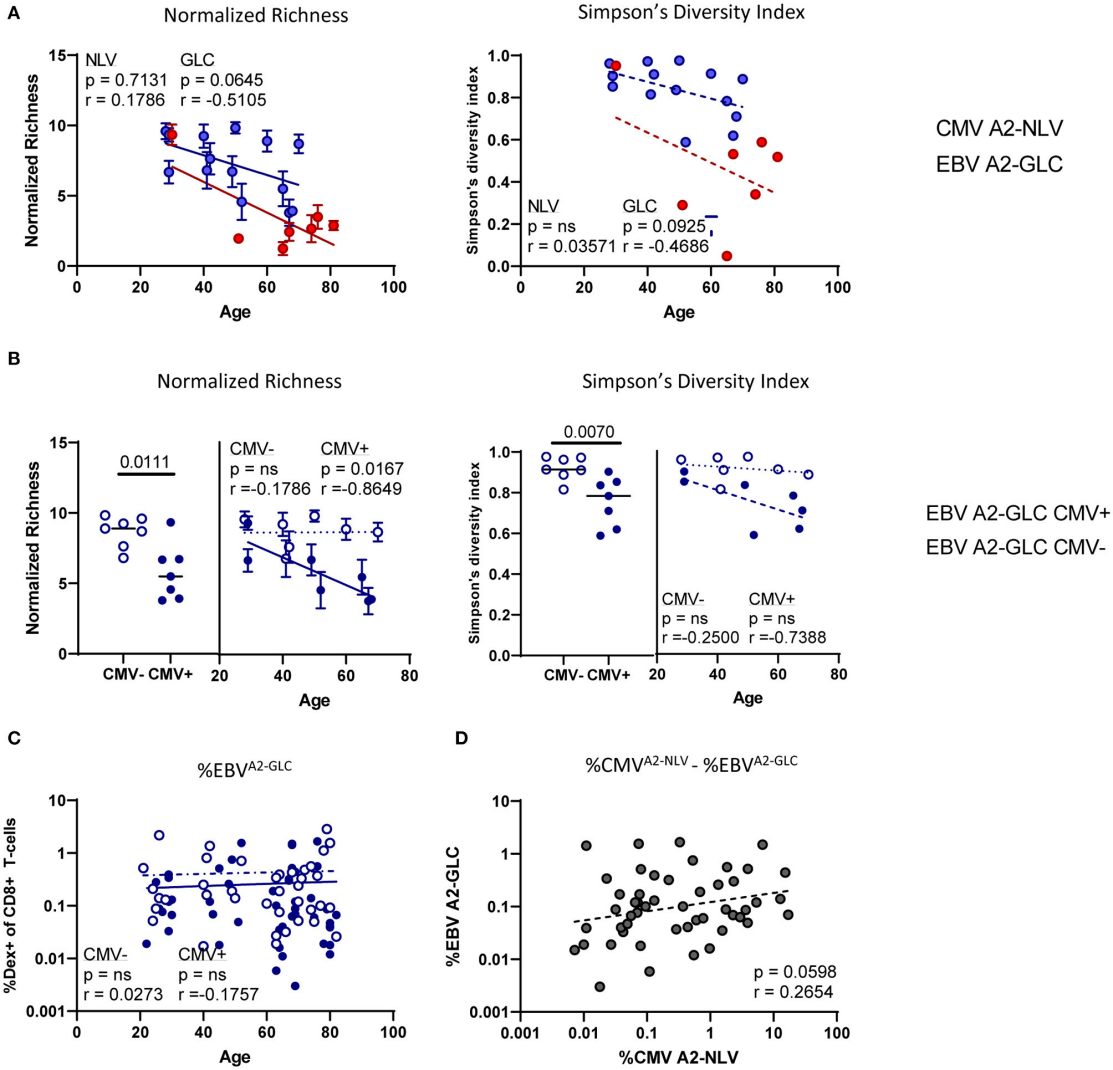
The CMV^A2−NLV^-specific TCRβ repertoire and the EBV^A2−GLC^-specific TCRβ repertoire decrease with age, whereas the diversity of the EBV^A2−GLC^-specific TCRβ repertoire is lower in the CMV^+^ individuals than in the CMV^−^ individuals. **(A)** Correlation plots of normalized richness score (left) and Simpson's diversity index (right panels) of CMV^A2−NLV^-specific and EBV^A2−GLC^-specific T-cell repertoires and age. Whiskers denote standard deviations over 1,000 subsampling iterations. **(B)** Normalized richness score and Simpson's diversity index of the EBV^A2−GLC^-specific T-cell repertoire divided in CMV^−^ (blue, open circles) and CMV^+^ individuals (blue, filled circles). **(C)** Percentage of EBV^A2−GLC^-specific CD8^+^ T cells in all CMV^−^ (blue open circles) and CMV^+^ individuals (blue filled circles). **(D)** Correlation of CMV^A2−NLV^ and EBV^A2−GLC^-specific CD8^+^ T-cell frequencies within donors. Frequencies are log transformed for statistical analysis. Solid lines indicate a slope significantly (*p* < 0.05) different from a slope of 0, whereas a dotted line indicates no significant difference. All correlations were tested by Spearman's Rank correlation coefficient.

To investigate whether age is associated with the diversity of the antigen-specific T-cell repertoire, we analyzed the normalized richness and the Simpson's diversity index of the repertoire of both CMV^A2−NLV^-specific and EBV^A2−GLC^-specific T cells in relation to the age of the individuals. The normalized richness of the antigen-specific T-cell repertoires against both CMV^A2−NLV^ and EBV^A2−GLC^ showed a negative trend with age (*p* = ns, *r* = 0.1786 and *p* = 0.0645, *r* = −0.5105, respectively), although the decrease with age observed for the CMV^A2−NLV^-specific samples was largely based on the datapoint of one young adult (Figure 5A). The Simpson's diversity index showed the same negative trend, although this was also not significant (*p* = ns for CMV^A2−NLV^ and *p* = 0.0925 for EBV^A2−GLC^) (Figure 5A). This suggests that the diversity of the CMV^A2−NLV^-specific and EBV^A2−GLC^-specific T-cell repertoire decrease with age.

As there are indications in mice that CMV-infection can affect the T-cell response against heterologous virus infections (Cicin-Sain et al., 2012; Mekker et al., 2012; Redeker et al., 2017), we then stratified the EBV^A2−GLC^-specific T-cell data according to the individuals' CMV status. We found a higher diversity of the EBV^A2−GLC^-specific T-cell repertoire in CMV^−^ compared to CMV^+^ individuals based on both normalized richness (*p* = 0.0111) and Simpson's diversity index (*p* = 0.0070) (Figure 5B). Linear regression analysis of these data suggests a lower diversity of the EBV^A2−GLC^-specific T-cell repertoire in the presence of CMV over the entire observed age range (Figure 5B). A significant decrease in diversity of the EBV^A2−GLC^-specific T-cell repertoire with age was only observed in CMV^+^ individuals.

A likely explanation for the decreased diversity of the EBV^A2−GLC^-specific T-cell repertoire in CMV^+^ individuals could be memory attrition, i.e., large frequencies of CMV^A2−NLV^-specific T cells outcompeting non-CMV-specific T cells. If there is indeed a role for memory attrition, one would expect that (1) the percentage of EBV^A2−GLC^-specific T cells is lower in CMV^+^ compared to CMV^−^ individuals, and (2) that the percentage of EBV^A2−GLC^-specific T cells correlates negatively with the percentage of CMV^A2−NLV^-specific T cells. CMV^+^ individuals indeed had significantly lower percentages of EBV^A2−GLC^-specific CD8^+^ T cells compared to CMV^−^ individuals (*p* = 0.0300) (Supplementary Figure 6A). The lower fraction of EBV^A2−GLC^-specific T cells was seen at all ages (Figure 5C). However, the percentage of EBV-specific T cells was not negatively associated with the percentage of CMV^A2−NLV^-specific T cells. If anything, there was a trend toward a positive correlation between both antigen-specific T-cell frequencies (*p* = 0.0598, *r*^2^ = 0.2654) (Figure 5D). Thus, the decreased frequency and diversity of EBV^A2−GLC^-specific T cells in CMV^+^ individuals does not seem to be due to memory attrition.

Based on the same t-SNE cluster analysis as shown in Figure 1B, we compared the EBV^A2−GLC^-specific CD8^+^ T-cell population of CMV^−^ (*n* = 32) and CMV^+^ (*n* = 40) individuals (Supplementary Figure 6B). For this analysis, data from donors of all ages were pooled. For none of the three earlier identified clusters did we find any significant differences between CMV^+^ and CMV^−^ individuals (Supplementary Figure 6B). As the t-SNE gates were rather rough, we wondered whether there would be any differences in the phenotype of EBV^A2−GLC^-specific T cells when analyzing the data in more detail. Based on conventional gating, we observed no substantial CMV-related differences based on for example the percentage of CD57^+^ or KLRG-1^+^ cells. Only the percentage of PD-1^+^ expressing EBV^A2−GLC^-specific T cells was significantly higher in CMV^−^ as compared to CMV^+^ individuals (*p* = 0.0255), while the EBV^A2−GLC^-specific T-cell population in CMV^+^ individuals had a significantly higher percentage of effector memory T cells than in CMV^−^ individuals (*p* = 0.0106) (Supplementary Figure 6C). Thus, CMV-infection may induce subtle changes in non-CMV-specific T cell populations, like those specific for EBV.

## Discussion

In this study, we investigated the effect of age and CMV-infection on the phenotype and diversity of the antigen-specific T-cell repertoire. We focused on CMV^A2−NLV^-specific and EBV^A2−GLC^-specific T cells, as these antigen-specific T cells are readily detectable in the T-cell pool at all ages. The antigen-specific T cells against both persistent viruses showed an age-related increase in the expression of several markers associated with a more differentiated phenotype, including KLRG-1, an increase in the fraction of terminally differentiated T cells and a decrease in the diversity of the antigen-specific T-cell repertoire. CMV-infection has also been proposed to reduce the diversity of the total memory T-cell pool (Khan et al., 2002; Emerson et al., 2017). However, the effect of CMV on the diversity of other antigen-specific T-cell repertoires remains poorly understood. Here we show that CMV infection is associated with a lower diversity of the EBV^A2−GLC^-specific T-cell repertoire. Although the exact mechanism behind this association remains unknown, our data suggest that the decreased diversity of the EBV^A2−GLC^-specific T-cell repertoire in CMV^+^ individuals is not due to memory attrition. We found that antigen-specific T cells against CMV^A2−NLV^ and EBV^A2−GLC^ are different at the phenotypic level; CMV^A2−NLV^-specific T cells have higher percentages of Temra cells, as defined by CD27- and CD45RO-, and higher expression of CD57 than EBV^A2−GLC^-specific T cells. These findings are in line with other studies, showing that EBV^A2−GLC^-specific T cells are predominantly CD45RO^+^ (Kuijpers et al., 2003) and more often express CD27 (Appay et al., 2002). The presence of terminally differentiated CMV^A2−NLV^-specific T cells is probably explained by the presence of large clonal expansions, which are typical for CMV infection (van den Berg et al., 2019).

The relatively high expression of markers associated with a more differentiated phenotype and the relatively low TCR repertoire diversity of the CMV^A2−NLV^-specific T-cell population becomes even more pronounced in individuals at older age. Based on the high frequencies of CMV^A2−NLV^-specific T cells in older adults, this age-effect is probably also linked to the presence of large clonal expansions consisting of terminally differentiated cells. It has been suggested that the increase in CMV-specific T-cell numbers with age is due to periodical infectious reactivation (van Boven et al., 2017). Although EBV^A2−GLC^-specific T cells also show changes associated with age, these are less pronounced than for CMV^A2−NLV^-specific T cells. This may be related to differences in cellular tropism between the two herpes viruses (Shenk, 2008; Hatton et al., 2014), or to possible differences in viral reactivation frequencies (Scheinberg et al., 2007; Thomasini et al., 2017).

It is tempting to speculate that the features of the T-cell responses against CMV^A2−NLV^ and EBV^A2−GLC^ that we observed are characteristic for the immune response to these two viruses in general. Although these two epitopes tend to be immunodominant, and thereby represent a fair share of the T-cell response in many individuals, it was recently shown that different combinations of HLA-alleles can influence the immunodominance of an epitope (Maleeva et al., 2019). This may also explain the large variation in antigen-specific T-cell frequencies that we observed in the four age groups. It remains to be investigated whether our results also apply to other epitopes for these two viruses, and to individuals in which these responses are less dominant.

Previous studies on the effect of aging on the antigen-specific T-cell repertoire have mostly focused on the maintenance of TCR sequences that are shared between individuals or that occur at different timepoints (Annels et al., 2000; Klarenbeek et al., 2012) or on a biased usage of Vβ-segments (Schwanninger et al., 2008). As these studies observed dominant T-cell clones both in a longitudinal setting and in a cross-sectional setting across different ages, they led to the view that the antigen-specific T-cell repertoires against CMV and EBV are relatively stable over time. Even though the T-cell repertoire analyses were performed on a relatively small number of samples, our direct assessment of the TCR diversity of EBV^A2−GLC^-specific T cells, and to a lesser extent of CMV^A2−NLV^-specific T cells, showed a clear decrease in diversity with age, suggesting that the antigen-specific T-cell repertoires against these viruses are not as stable as previously thought. These seemingly contradicting conclusions may be due to the process of convergent contraction of the T cell repertoire, in which lower frequency clonotypes are lost over time, while only few T cells persist (Smith et al., 2020). Observations of these persisting T-cell clones over time would suggest that the antigen-specific T-cell repertoire is relatively stable, even though the richness and diversity of the repertoire may decrease with age. To study whether convergent contraction of the T-cell repertoire is indeed happening, a longitudinal study should be performed, focusing on the richness and diversity of the antigen-specific T-cell repertoire.

It remains unknown *why* the diversity of antigen-specific T-cell repertoires decreases with age and whether age is the real driver of the decrease in T-cell diversity or whether other factors play an important role. We cannot exclude the possibility that the older individuals of the study population had been infected at an older age, possibly leading to an antigen-specific T-cell repertoire of lower diversity because the diversity of the naive (precursor) pool is known to decrease with age (Britanova et al., 2014; Egorov et al., 2018). However, the recent finding that only a very small percentage of individuals seroconvert for CMV at later age (Samson et al., 2020), as well as the finding that more than 90% of the population is infected with EBV during adolescence (Balfour et al., 2013; Winter et al., 2020), makes this explanation unlikely. In our view, a more likely explanation for the reduced diversity in the antigen-specific T-cell repertoire of older individuals would be that older individuals have been infected for a longer time, and have lost more T-cell clones over time, for example due to exhaustion after restimulation (Lanfermeijer et al., 2020).

Our results indicate that CMV-infection is associated with a lower diversity of the EBV^A2−GLC^-specific T-cell repertoire. As it is generally thought that T-cell receptor diversity is positively correlated with the level of protection against infectious diseases, one would expect that this decreased diversity would lead to a decreased EBV-specific T-cell response in CMV^+^ individuals. If our findings also hold true for other antigens, this would suggest that CMV^+^ individuals are less protected against other infections. This is in line with most mouse studies, which show a negative effect of CMV-infection on the T-cell efficacy against heterologous infections (Cicin-Sain et al., 2012; Mekker et al., 2012; Smithey et al., 2012; Redeker et al., 2017). *How* CMV-infection would lead to this lower diversity remains unknown. Although memory attrition has often been suggested to play a role, our data do not support this idea, as we observed a positive correlation between the frequencies of EBV^A2−GLC^- and CMV^A2−NLV^-specific T cells. The explanation for this positive correlation might be that some individuals are better T-cell responders than others. The observation that CMV-infection leads to lower frequencies of antigen-specific T cells is not surprising, as it is a relative measure, which is easily skewed by the high percentages of CMV-specific T cells. Likewise, in another study of our group it was shown that influenza-specific T-cell frequencies in older individuals were lower in CMV^+^ compared to CMV^−^ individuals. Importantly, however, this did not result in lower influenza-specific IFNγ responses in CMV^+^ individuals (van den Berg et al., in press), suggesting that CMV did not negatively impact the influenza-specific T-cell response; although sample sizes were unfortunately too small to confirm this based on the diversity of the influenza-specific TCR repertoire. It remains puzzling why these different results are observed between studies, and it would be interesting to understand whether e.g., the acute or chronic nature of a pathogen plays a role.

T cells isolated from blood represent only a small fraction of the total T-cell pool in the body, as it has been estimated that blood contains only 2 percent of all the T cells in the body (Westermann, 1990). Although many T cells travel through different compartments in the body, it remains to be investigated whether antigen-specific T-cell characteristics, like their phenotype and repertoire diversity, that are observed in the blood can be extrapolated to T cells in other sites of the body. The presence of tissue-resident T cells, which hardly circulate through the blood, complicates this even further. In the case of CMV and EBV, however, the blood may in fact be the most informative site to follow the antigen-specific T-cell response. It has previously been suggested that most CMV-specific T cells are present in the blood (Gordon et al., 2017). In line with this, a recent human study showed that most terminally differentiated memory CD8^+^ T cells, including CMV-specific T cells, are confined to the intravascular circulation and do not circulate through the thoracic duct lymph (Buggert et al., 2020). Also for EBV, a bloodborne virus infecting B cells, the blood may in fact be the most representative compartment to study the antigen-specific T-cell response. It remains to be investigated whether the changes we observed in the antigen-specific T-cell repertoire in the blood also apply to antigen-specific T cells in other sites of the body. So far, studies have shown both a minimal overlap in the naïve T-cell pool between spleen and lymph nodes (Thome et al., 2016), as well as high degrees of overlap when focusing on the memory T-cell pool between blood and the thoracic duct lymph (Buggert et al., 2020), between peripheral blood and lymph nodes (Remmerswaal et al., 2015) and between spleen and lymph nodes (Thome et al., 2014).

In contrast to the commonly-held view that the antigen-specific T-cell repertoires against CMV and EBV are relatively stable with age, we here show that they both clearly decrease with age. Our data suggest that not only age but also CMV-infection is associated with the diversity of the EBV^A2−GLC^-specific T-cell repertoire. Insights into how antigen-specific T-cell repertoires evolve with age and under the influence of other infections, like latent CMV, are important for the development of novel vaccination strategies to protect older adults against infectious diseases. One of the proposed strategies to prevent older adults is to induce protective immune responses through vaccination at a younger age. This would require stability of the induced immune response in order to provide protection later in life. Our data suggest that the antigen-specific T-cell repertoire is not as stable as previously thought. Unfortunately, this implies that vaccination may also come too early. Ideally, one would like to vaccinate before the age-associated decline in naive T-cell repertoire diversity, but late enough to ensure that a substantial level of protection is maintained until later in life. Further research is needed to investigate why some cells are maintained while others are not, and to define the optimal moment of vaccination to protect the elderly.

## Data Availability Statement

The raw data supporting the conclusions of this article will be made available by the authors, without undue reservation.

## Ethics Statement

The studies involving human participants were reviewed and approved by Central Committee on Research Involving Human Subjects of the Netherlands and ethical committee, METC Noord Holland. The patients/participants provided their written informed consent to participate in this study.

## Author Contributions

The original idea for this study was from DvB and JAMB. JL performed the majority of experiments, gathered data, and analysis. PdG performed data analysis. MH and MV performed experiments. JvB designed the original studies of the individuals. DvB and JAMB supervised the project. JL and PdG prepared figures and wrote manuscript with contributions and review from DvB, JAMB, and JvB. All authors contributed to the article and approved the submitted version.

## Conflict of Interest

The authors declare that the research was conducted in the absence of any commercial or financial relationships that could be construed as a potential conflict of interest.

## References

[B1] Almanzar G. Schwaiger S. Jenewein B. Keller M. Herndler-Brandstetter D. Wurzner R.. (2005). Long-term cytomegalovirus infection leads to significant changes in the composition of the CD8+ T-cell repertoire, which may be the basis for an imbalance in the cytokine production profile in elderly persons. J. Virol. 79, 3675–3683. 10.1128/JVI.79.6.3675-3683.2005 15731261 PMC1075718

[B2] Annels N. E. Callan M. F. C. Tan L. Rickinson A. B. (2000). Changing patterns of dominant tcr usage with maturation of an EBV-specific cytotoxic T cell response. J. Immunol. 165, 4831–4841. 10.4049/jimmunol.165.9.4831 11046006

[B3] Appay V. Dunbar P. R. Callan M. Klenerman P. Gillespie G. M. A. Papagno L.. (2002). Memory CD8+ T cells vary in differentiation phenotype in different persistent virus infections. Nat. Med. 8, 379–385. 10.1038/nm0402-379 11927944

[B4] Balfour H. H. Jr Sifakis F. Sliman J. A. Knight J. A. Schmeling D. O. Thomas W. (2013). Age-specific prevalence of Epstein-Barr virus infection among individuals aged 6-19 years in the United States and factors affecting its acquisition. J. Infect. Dis. 208, 1286–1293. 10.1093/infdis/jit321 23868878

[B5] Briceno O. Lissina A. Wanke K. Afonso G. von Braun A. Ragon K.. (2016). Reduced naive CD8(+) T-cell priming efficacy in elderly adults. Aging Cell 15, 14–21. 10.1111/acel.12384 26472076 PMC4717282

[B6] Britanova O. V. Putintseva E. V. Shugay M. Merzlyak E. M. Turchaninova M. A. Staroverov D. B.. (2014). Age-related decrease in TCR repertoire diversity measured with deep and normalized sequence profiling. J. Immunol. 192, 2689–2698. 10.4049/jimmunol.1302064 24510963

[B7] Britanova O. V. Shugay M. Merzlyak E. M. Staroverov D. B. Putintseva E. V. Turchaninova M. A.. (2016). Dynamics of individual T cell repertoires: from cord blood to centenarians. J. Immunol. 196, 5005–5013. 10.4049/jimmunol.1600005 27183615

[B8] Buggert M. Vella L. A. Nguyen S. Wu V. H. Chen Z. Sekine T.. (2020). The identity of human tissue-emigrant CD8(+) T cells. Cell 183, 1946–1961. 10.1101/2020.08.11.236372 33306960 PMC9341432

[B9] Cardenas Sierra D. Velez Colmenares G. Orfao de Matos A. Fiorentino Gomez S. Quijano Gomez S. M. (2014). Age-associated Epstein-Barr virus-specific T cell responses in seropositive healthy adults. Clin. Exp. Immunol. 177, 320–332. 10.1111/cei.12337 24666437 PMC4089182

[B10] Chidrawar S. Khan N. Wei W. McLarnon A. Smith N. Nayak L.. (2009). Cytomegalovirus-seropositivity has a profound influence on the magnitude of major lymphoid subsets within healthy individuals. Clin. Exp. Immunol. 155, 423–432. 10.1111/j.1365-2249.2008.03785.x 19220832 PMC2669518

[B11] Cicin-Sain L. Brien J. D. Uhrlaub J. L. Drabig A. Marandu T. F. Nikolich-Zugich J. (2012). Cytomegalovirus infection impairs immune responses and accentuates T-cell pool changes observed in mice with aging. PLoS Pathog. 8:e1002849. 10.1371/journal.ppat.1002849 22916012 PMC3420928

[B12] Deng Y. Jing Y. Campbell A. E. Gravenstein S. (2004). Age-related impaired type 1 T cell responses to influenza: reduced activation ex vivo, decreased expansion in CTL culture in vitro, and blunted response to influenza vaccination in vivo in the elderly. J. Immunol. 172, 3437–3446. 10.4049/jimmunol.172.6.3437 15004143

[B13] Derhovanessian E. Larbi A. Pawelec G. (2009). Biomarkers of human immunosenescence: impact of Cytomegalovirus infection. Curr. Opin. Immunol. 21, 440–445. 10.1016/j.coi.2009.05.012 19535233

[B14] Egorov E. S. Kasatskaya S. A. Zubov V. N. Izraelson M. Nakonechnaya T. O. Staroverov D. B.. (2018). The changing landscape of naive T cell receptor repertoire with human aging. Front. Immunol. 9:1618. 10.3389/fimmu.2018.01618 30087674 PMC6066563

[B15] Elhanati Y. Sethna Z. Callan C. G. Jr. Mora T. Walczak A. M. (2018). Predicting the spectrum of TCR repertoire sharing with a data-driven model of recombination. Immunol. Rev. 284, 167–179. 10.1111/imr.12665 29944757 PMC6033145

[B16] Emerson R. O. DeWitt W. S. Vignali M. Gravley J. Hu J. K. Osborne E. J.. (2017). Immunosequencing identifies signatures of cytomegalovirus exposure history and HLA-mediated effects on the T cell repertoire. Nat. Genet. 49, 659–665. 10.1038/ng.3822 28369038

[B17] Gerritsen B. Pandit A. Andeweg A. C. de Boer R. J. (2016). RTCR: a pipeline for complete and accurate recovery of T cell repertoires from high throughput sequencing data. Bioinformatics 32, 3098–3106. 10.1093/bioinformatics/btw339 27324198 PMC5048062

[B18] Gordon C. L. Miron M. Thome J. J. Matsuoka N. Weiner J. Rak M. A.. (2017). Tissue reservoirs of antiviral T cell immunity in persistent human CMV infection. J. Exp. Med. 214, 651–667. 10.1084/jem.20160758 28130404 PMC5339671

[B19] Goronzy J. J. Fulbright J. W. Crowson C. S. Poland G. A. O'Fallon W. M. Weyand C. M. (2001). Value of immunological markers in predicting responsiveness to influenza vaccination in elderly individuals. J. Virol. 75, 12182–12187. 10.1128/JVI.75.24.12182-12187.2001 11711609 PMC116115

[B20] Hadrup S. R. Strindhall J. Kollgaard T. Seremet T. Johansson B. Pawelec G.. (2006). Longitudinal studies of clonally expanded CD8 T cells reveal a repertoire shrinkage predicting mortality and an increased number of dysfunctional cytomegalovirus-Specific T cells in the very elderly. J. Immunol. 176, 2645–2653. 10.4049/jimmunol.176.4.2645 16456027

[B21] Hatton O. L. Harris-Arnold A. Schaffert S. Krams S. M. Martinez O. M. (2014). The interplay between Epstein-Barr virus and B lymphocytes: implications for infection, immunity, and disease. Immunol. Res. 58, 268–276. 10.1007/s12026-014-8496-1 24619311 PMC4199828

[B22] Hoji A. Connolly N. C. Buchanan W. G. Rinaldo C. R. Jr. (2007). CD27 and CD57 expression reveals atypical differentiation of human immunodeficiency virus type 1-specific memory CD8+ T cells. Clin. Vaccine Immunol. 14, 74–80. 10.1128/CVI.00250-06 17079436 PMC1797708

[B23] Iancu E. M. Corthesy P. Baumgaertner P. Devevre E. Voelter V. Romero P.. (2009). Clonotype selection and composition of human CD8 T cells specific for persistent herpes viruses varies with differentiation but is stable over time. J. Immunol. 183, 319–331. 10.4049/jimmunol.0803647 19542443

[B24] Jubel J. M. Barbati Z. R. Burger C. Wirtz D. C. Schildberg F. A. (2020). The role of PD-1 in acute and chronic infection. Front. Immunol. 11:487. 10.3389/fimmu.2020.00487 32265932 PMC7105608

[B25] Khan N. Hislop A. Gudgeon N. Cobbold M. Khanna R. Nayak L.. (2004). Herpesvirus-specific CD8 T cell immunity in old age: cytomegalovirus impairs the response to a coresident EBV infection. J. Immunol. 173, 7481–7489. 10.4049/jimmunol.173.12.7481 15585874

[B26] Khan N. Shariff N. Cobbold M. Bruton R. Ainsworth J. A. Sinclair A. J.. (2002). Cytomegalovirus seropositivity drives the CD8 t cell repertoire toward greater clonality in healthy elderly individuals. J. Immunol. 169, 1984–1992. 10.4049/jimmunol.169.4.1984 12165524

[B27] Klarenbeek P. L. Remmerswaal E. B. ten Berge I. J. Doorenspleet M. E. van Schaik B. D. Esveldt R. E.. (2012). Deep sequencing of antiviral T-cell responses to HCMV and EBV in humans reveals a stable repertoire that is maintained for many years. PLoS Pathog. 8:e1002889. 10.1371/journal.ppat.1002889 23028307 PMC3460621

[B28] Kotecha N. Krutzik P. O. Irish J. M. (2010). Web-based analysis and publication of flow cytometry experiments. Curr. Protoc. Cytom. 10:17. 10.1002/0471142956.cy1017s53 20578106 PMC4208272

[B29] Kuijpers T. W. Vossen M. T. Gent M. R. Davin J. C. Roos M. T. Wertheim-van Dillen P. M.. (2003). Frequencies of circulating cytolytic, CD45RA+CD27-, CD8+ T lymphocytes depend on infection with CMV. J. Immunol. 170, 4342–4348. 10.4049/jimmunol.170.8.4342 12682271

[B30] Lanfermeijer J. Borghans J. A. M. van Baarle D. (2020). How age and infection history shape the antigen-specific CD8(+) T-cell repertoire: implications for vaccination strategies in older adults. Aging Cell 19:e13262. 10.1111/acel.13262 33078890 PMC7681067

[B31] Lindau P. Mukherjee R. Gutschow M. V. Vignali M. Warren E. H. Riddell S. R.. (2019). Cytomegalovirus exposure in the elderly does not reduce CD8 T cell repertoire diversity. J. Immunol. 202, 476–483. 10.4049/jimmunol.1800217 30541882 PMC6321841

[B32] Maleeva A. V. Shmarov V. A. Kiryukhin D. O. Efimov G. A. Savchenko V. G. (2019). Repertoire of cytomegalovirus-specific T cells is focused on the immunodominant epitopes in fixed hierarchy dependent on HLA genotype of the donor. Blood 134:2327. 10.1182/blood-2019-130241

[B33] Market E. Papavasiliou F. N. (2003). V(D)J recombination and the evolution of the adaptive immune system. PLoS Biol. 1:E16. 10.1371/journal.pbio.0000016 14551913 PMC212695

[B34] Mekker A. Tchang V. S. Haeberli L. Oxenius A. Trkola A. Karrer U. (2012). Immune senescence: relative contributions of age and cytomegalovirus infection. PLoS Pathog. 8:e1002850. 10.1371/journal.ppat.1002850 22916013 PMC3420944

[B35] Nikolich-Zugich J. (2008). Ageing and life-long maintenance of T-cell subsets in the face of latent persistent infections. Nat. Rev. Immunol. 8, 512–522. 10.1038/nri2318 18469829 PMC5573867

[B36] Pawelec G. Akbar A. Caruso C. Grubeck-Loebenstein B. Solana R. Wikby A. (2005). Human immunosenescence: is it infectious? Immunol. Rev. 205, 257–268. 10.1111/j.0105-2896.2005.00271.x 15882359

[B37] Qi Q. Liu Y. Cheng Y. Glanville J. Zhang D. Lee J. Y.. (2014). Diversity and clonal selection in the human T-cell repertoire. Proc. Natl. Acad. Sci. U. S. A. 111, 13139–13144. 10.1073/pnas.1409155111 25157137 PMC4246948

[B38] Redeker A. Remmerswaal E. B. M. van der Gracht E. T. I. Welten S. P. M. Hollt T. Koning F.. (2017). The contribution of cytomegalovirus infection to immune senescence is set by the infectious dose. Front. Immunol. 8:1953. 10.3389/fimmu.2017.01953 29367854 PMC5768196

[B39] Remmerswaal E. B. Klarenbeek P. L. Alves N. L. Doorenspleet M. E. van Schaik B. D. Esveldt R. E.. (2015). Clonal evolution of CD8+ T cell responses against latent viruses: relationship among phenotype, localization, and function. J. Virol. 89, 568–580. 10.1128/JVI.02003-14 25339770 PMC4301136

[B40] Rosendahl Huber S. K. Hendriks M. Jacobi R. H. J. van de Kassteele J. Mandersloot-Oskam J. C. van Boxtel R. A. J.. (2018). Immunogenicity of influenza vaccines: evidence for differential effect of secondary vaccination on humoral and cellular immunity. Front. Immunol. 9:3103. 10.3389/fimmu.2018.03103 30761157 PMC6362424

[B41] Sad S. Krishnan L. (2003). Maintenance and attrition of T-cell memory. Crit. Rev. Immunol. 23, 129–147. 10.1615/CritRevImmunol.v23.i12.70 12906263

[B42] Samson L. D. van den Berg S. P. Engelfriet P. Boots A. M. Hendriks M. de Rond L. G.. (2020). Limited effect of duration of CMV infection on adaptive immunity and frailty: insights from a 27-year-long longitudinal study. Clin. Transl. Immunol. 9:e1193. 10.1002/cti2.1193 33133599 PMC7586993

[B43] Scheinberg P. Fischer S. H. Li L. Nunez O. Wu C. O. Sloand E. M.. (2007). Distinct EBV and CMV reactivation patterns following antibody-based immunosuppressive regimens in patients with severe aplastic anemia. Blood 109, 3219–3224. 10.1182/blood-2006-09-045625 17148582 PMC1852232

[B44] Schwanninger A. Weinberger B. Weiskopf D. Herndler-Brandstetter D. Reitinger S. Gassner C.. (2008). Age-related appearance of a CMV-specific high-avidity CD8+ T cell clonotype which does not occur in young adults. Immun. Ageing 5:14. 10.1186/1742-4933-5-14 19014475 PMC2596076

[B45] Sethna Z. Elhanati Y. Callan C. G. Walczak A. M. Mora T. (2019). OLGA: fast computation of generation probabilities of B- and T-cell receptor amino acid sequences and motifs. Bioinformatics 35, 2974–2981. 10.1093/bioinformatics/btz035 30657870 PMC6735909

[B46] Shenk T. E. S. M. F. (2008). Human cytomegalovirus. Curr. Top. Microbiol. Immunol. 325, 297–313. 10.1007/978-3-540-77349-8 18637497

[B47] Shugay M. Bagaev D. V. Zvyagin I. V. Vroomans R. M. Crawford J. C. Dolton G.. (2017). VDJdb: a curated database of T-cell receptor sequences with known antigen specificity. Nucleic Acids Res. 46, 419–427. 10.1093/nar/gkx760 28977646 PMC5753233

[B48] Shugay M. Britanova O. V. Merzlyak E. M. Turchaninova M. A. Mamedov I. Z. Tuganbaev T. R.. (2014). Towards error-free profiling of immune repertoires. Nat. Methods 11, 653–655. 10.1038/nmeth.2960 24793455

[B49] Smith C. J. Venturi V. Quigley M. F. Turula H. Gostick E. Ladell K.. (2020). Stochastic expansions maintain the clonal stability of CD8(+) T cell populations undergoing memory inflation driven by murine cytomegalovirus. J. Immunol. 204, 112–121. 10.4049/jimmunol.1900455 31818981 PMC6920548

[B50] Smithey M. J. Li G. Venturi V. Davenport M. P. Nikolich-Zugich J. (2012). Lifelong persistent viral infection alters the naive T cell pool, impairing CD8 T cell immunity in late life. J. Immunol. 189, 5356–5366. 10.4049/jimmunol.1201867 23087407 PMC3504138

[B51] Smithey M. J. Venturi V. Davenport M. P. Buntzman A. S. Vincent B. G. Frelinger J. A.. (2018). Lifelong CMV infection improves immune defense in old mice by broadening the mobilized TCR repertoire against third-party infection. Proc. Natl. Acad. Sci. U. S. A. 115, E6817–E6825. 10.1073/pnas.1719451115 29967140 PMC6055168

[B52] Sukdolak C. Tischer S. Dieks D. Figueiredo C. Goudeva L. Heuft H. G.. (2013). CMV-, EBV- and ADV-specific T cell immunity: screening and monitoring of potential third-party donors to improve post-transplantation outcome. Biol. Blood Marrow Transplant 19, 1480–1492. 10.1016/j.bbmt.2013.07.015 23891747

[B53] Tcherniaeva I. den Hartog G. Berbers G. van der Klis F. (2018). The development of a bead-based multiplex immunoassay for the detection of IgG antibodies to CMV and EBV. J. Immunol. Methods 462, 1–8. 10.1016/j.jim.2018.07.003 30056034

[B54] Thomasini R. L. Pereira D. S. Pereira F. S. M. Mateo E. C. Mota T. N. Guimaraes G. G.. (2017). Aged-associated cytomegalovirus and Epstein-Barr virus reactivation and cytomegalovirus relationship with the frailty syndrome in older women. PLoS ONE 12:e0180841. 10.1371/journal.pone.0180841 28700679 PMC5507269

[B55] Thome J. J. Grinshpun B. Kumar B. V. Kubota M. Ohmura Y. Lerner H.. (2016). Longterm maintenance of human naive T cells through *in situ* homeostasis in lymphoid tissue sites. Sci Immunol. 1:6506. 10.1126/sciimmunol.aah6506 28361127 PMC5367636

[B56] Thome J. J. Yudanin N. Ohmura Y. Kubota M. Grinshpun B. Sathaliyawala T.. (2014). Spatial map of human T cell compartmentalization and maintenance over decades of life. Cell 159, 814–828. 10.1016/j.cell.2014.10.026 25417158 PMC4243051

[B57] Tu W. Rao S. (2016). Mechanisms underlying T cell immunosenescence: aging and cytomegalovirus infection. Front. Microbiol. 7:2111. 10.3389/fmicb.2016.02111 28082969 PMC5186782

[B58] Turner S. J. La Gruta N. L. Kedzierska K. Thomas P. G. Doherty P. C. (2009). Functional implications of T cell receptor diversity. Curr. Opin. Immunol. 21, 286–290. 10.1016/j.coi.2009.05.004 19524428 PMC2706259

[B59] van Boven M. van de Kassteele J. Korndewal M. J. van Dorp C. H. Kretzschmar M. van der Klis F.. (2017). Infectious reactivation of cytomegalovirus explaining age- and sex-specific patterns of seroprevalence. PLoS Comput. Biol. 13:e1005719. 10.1371/journal.pcbi.1005719 28949962 PMC5630159

[B60] van den Berg S. P. H. Lanfermeijer J. Jacobi R. H. J. Hendriks M. Vos M. van Schuijlenburg R.. (in press). Latent CMV infection is associated with lower influenza virus-specific memory T-cell frequencies, but not with an impaired T-cell response to acute influenza virus infection. Front. Immunol. 10.3389/fimmu.2021.663664. PMC8131658 34025665

[B61] van den Berg S. P. H. Pardieck I. N. Lanfermeijer J. Sauce D. Klenerman P. van Baarle D.. (2019). The hallmarks of CMV-specific CD8 T-cell differentiation. Med. Microbiol. Immunol. 208, 365–373. 10.1007/s00430-019-00608-7 30989333 PMC6647465

[B62] Venturi V. Kedzierska K. Price D. A. Doherty P. C. Douek D. C. Turner S. J.. (2006). Sharing of T cell receptors in antigen-specific responses is driven by convergent recombination. PNAS 103, 18691–189696. 10.1073/pnas.0608907103 17130450 PMC1693724

[B63] Venturi V. Kedzierska K. Turner S. J. Doherty P. C. Davenport M. P. (2007). Methods for comparing the diversity of samples of the T cell receptor repertoire. J. Immunol. Methods. 321, 182–195. 10.1016/j.jim.2007.01.019 17337271

[B64] Venturi V. Price D. A. Douek D. C. Davenport M. P. (2008). The molecular basis for public Tcell responses? Nat. Rev. 8:2260. 10.1038/nri2260 18301425

[B65] Voisinne G. Gonzalez de Peredo A. Roncagalli R. (2018). CD5, an undercover regulator of TCR signaling. Front. Immunol. 9:2900. 10.3389/fimmu.2018.02900 30581443 PMC6292949

[B66] Westermann J. P. R. (1990). Lymphocyte subsets in the blood: a diagnostic window on the lymphoid system. Immunol. Today 11, 406–410. 10.1016/0167-5699(90)90160-B 2078294

[B67] Winter J. R. Jackson C. Lewis J. E. Taylor G. S. Thomas O. G. Stagg H. R. (2020). Predictors of Epstein-Barr virus serostatus and implications for vaccine policy: a systematic review of the literature. J. Glob. Health 10:010404. 10.7189/jogh.10.010404 32257152 PMC7125428

[B68] Yoshida K. Cologne J. B. Cordova K. Misumi M. Yamaoka M. Kyoizumi S.. (2017). Aging-related changes in human T-cell repertoire over 20 years delineated by deep sequencing of peripheral T-cell receptors. Exp. Gerontol. 96, 29–37. 10.1016/j.exger.2017.05.015 28535950

