# Supplementary material for: Age and CMV-Infection Jointly Affect the EBV-Specific CD8+ T-Cell Repertoire
Source: Front Aging. 2021 Apr 29;2:665637. doi: 10.3389/fragi.2021.665637 (PMC9261403; doi:10.3389/fragi.2021.665637)
Supplement: Supplementary file 1 [file Data_Sheet_1.pdf]

Supplement: Cleaning sequence data

Introduction

Since we analyzed antigen-specific subsets of the T-cell repertoire, the samples consisted of relatively small numbers of T cells. This may cause problems when applying standard methods for sequencing and analyzing T-cell repertoire diversity. To overcome these issues, we applied various filtering steps to minimize the potential impact of unavoidable experimental errors on the results presented in the main text.

An important step in many TCR-analysis protocols is the use of unique molecular identifiers (UMIs). These are introduced before extensive multiplication by PCR and hence allow to map PCR-products to their ancestral cDNA-molecule. This way, one can correct for biases by uneven PCR amplification and other errors that are likely to arise in some of the sequences. While removing biases, the results may still be affected by two other factors. The first is that if a sample contains a very abundant UMI-TCR pair (which originated from just one cDNA molecule), even the smallest contamination between samples may cause this UMI-TCR pair to also occur in another sample. This shared TCR sequence would then be interpreted as a clonotype present in multiple individuals. The other problem is that during PCR and sequencing of the product, errors may be introduced in the UMI sequence. This will lead to an inflated number of distinct UMI sequences that are observed with a given TCR, and hence to an overestimation of the abundance of individual TCR sequences in a sample.

When analyzing samples containing millions of unsorted T cells one may accept these factors to play a role, as their relative impact is expected to be small. However, since the number of sequences in our samples is limited, small biases could have a major impact on the results. Therefore, we performed additional filtering steps to make sure that our results are not affected by these confounding factors. We did this in a step-wise approach by using the abundance, sharing and nucleotide sequence of the UMIs.

A) Pairing, UMI-identification and V/J-alignment

Demultiplexed samples were first merged using tool Paired-End reAd mergeR (PEAR, Zhang Bioinformatics 2014). Since assembly efficiency was variable between different samples, we decided to also include the non-assembled reverse read in the analysis. The 12nt UMI sequences in the reads were identified using the 'Checkout' algorithm in Recover T Cell Receptor (RTCR, Gerritsen Bioinformatics 2016). For all samples together, 1.06 million UMI sequences could be found in a total of 34.3 million reads. Within each sample, the reads were then collapsed by their UMI into consensus sequences using the 'umi_group_ec' method of RTCR. We then used the 'run' function of RTCR to align the sequences to the reference TRBV and TRBJ genes. Only the alignment information was used, ignoring the further clustering steps that RTCR performs by default. We proceeded with the ~ 34% UMI-based consensus sequences in which the V as well as the J gene could be identified.

B) Within-sample cleaning

Since we separately took the merged and non-assembled reverse reads into account, individual UMIs could generate two consensus sequences. We confirmed that in the vast majority of such cases, where both V and J genes could be identified, these were identical. We selected the consensus sequence with highest support, i.e., based on the highest number of reads in such cases. Next, we removed all UMIs that were observed in just a single read, because they are most likely erroneous, leaving $6.6*10^4$ consensus sequences for all samples together. We then analyzed Hamming Distances between UMI sequences observed within and between samples. This showed that there were about 50 times more

similar (measured as Hamming Distance ≤ 3) UMI pairs within than between samples. We noticed that consensus sequences of similar UMIs within a sample had identical V and/or J genes in most cases. This indicates mutation of UMI sequences, which would lead to an inflated abundance estimation of the corresponding TCR sequences. We corrected for this by clustering pairs of UMIs within a Hamming Distance of 3, and subsequently removing the UMIs with the lowest read counts. This approach excluded many likely UMI-mutants that were supported by a much lower number of reads (mean 19) than the remaining sequences ($2.0*10^3$). RTCR was run individually on each of the remaining $7.1*10^3$ UMI-based consensus sequences to identify their CDR3 sequence while also retaining the UMI sequence.

C) Cross-sample cleaning

A substantial fraction of the UMIs (21%) was observed in more than one sample. Although overlap of UMIs between samples is theoretically possible, this was observed much more often than expected by chance, suggesting cross-sample contamination. Indeed, 93% of the UMIs that were shared between at least two samples showed identical CDR3 amino acid sequences in the corresponding TCRB sequences. We reasoned that contamination by abundant UMI-TCR pairs is expected to be represented by the same UMI-TCR pair at a much lower frequency in other samples. For less abundant UMI-TCR pairs, the frequency differences between samples are smaller, making it often impossible to tell which sample contained the genuine UMI-TCR pair and which the contamination. Hence, we only accepted overlapping UMIs between samples if 1) the frequency of the UMI in one of the samples was more than 1000 reads and 2) the frequency of the UMI in the other sample was more than 10% of the maximum frequency. The few overlapping UMIs that hence remained, all had different CDR3 sequences in both samples, suggesting that they were not due to contamination. We proceeded with these remaining overlapping UMI-TCR pairs and all others that were not shared between samples.

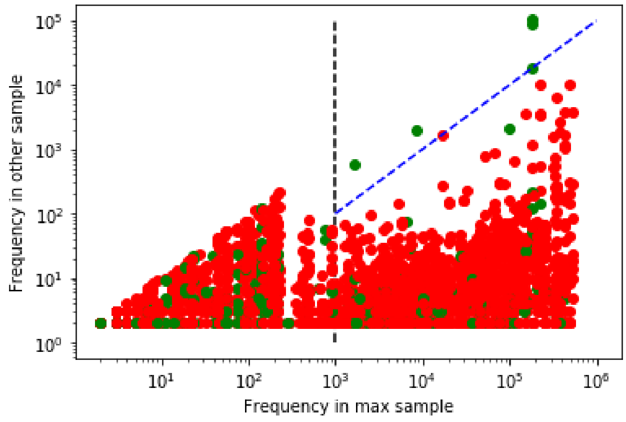

Figure SA: Read counts of UMI sequences that are shared between samples. Red dots indicate pairs with identical UMI and CDR3 sequence in two samples (indicating contamination), green dots represent pairs with identical UMI but different CDR3 sequence. The vertical black dashed line represents the read count threshold of 1000, below which all sequences with the corresponding UMI are deleted. The diagonal blue line indicates the 10% of maximum frequency threshold. UMI duplicates above this threshold are both kept (upper right quadrant), below the threshold only the UMI with maximum number of reads is accepted (lower right quadrant).

D) Read count threshold

Although we applied within-sample UMI-clustering and between-sample cleaning of UMI-overlap, still erroneous sequences could have remained, for example by mutation of UMI sequences that arose by contamination. Indeed, there were multiple samples containing abundant UMI-CDR3 pairs and also many identical CDR3 sequences with different UMIs. Most of these other UMIs were supported by

only few reads. By inspecting individual samples as well as all samples together, we often observed a bimodal distribution of read counts. Assuming that the UMIs supported by fewer reads are enriched for erroneous sequences, we only used UMI-CDR3 pairs above a read count threshold. We decided to only keep UMI-CDR3 pairs supported by at least 40 reads to further exclude confounding effects in our data. Note that we also tested the effect of an even stricter threshold of 600 reads, but this did not change our results qualitatively. The distributions of UMI distances within and between samples indicates that our cleaning procedure was successful.

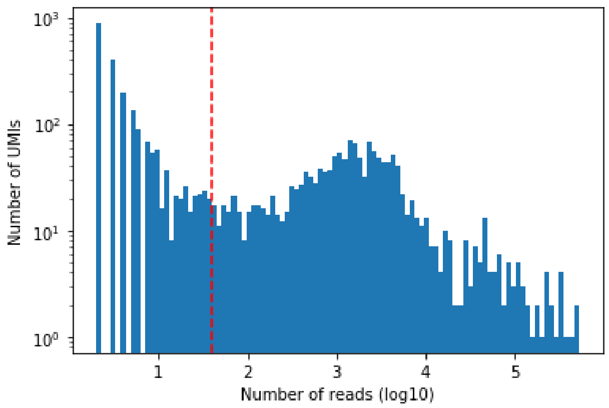

Figure SB: Histogram of all UMI read counts in each sample. The minimum read count threshold (40) is indicated with the horizontal red line.

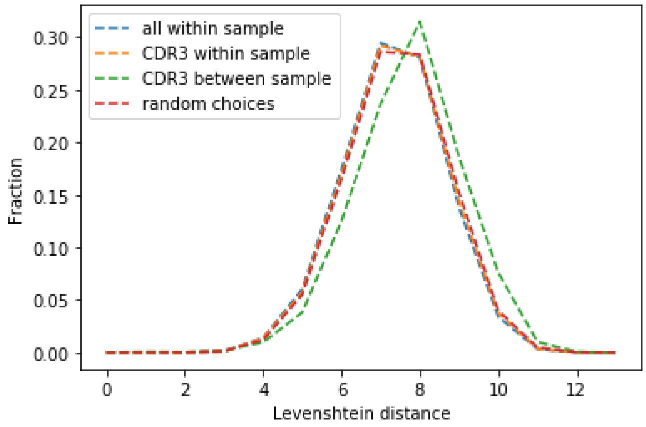

Figure SC: Distributions of UMI distances after data cleaning. Pairwise comparison between sets of UMI sequences using Levenshtein Distance. Blue: all UMIs are compared to all other UMIs in the same sample. Orange: UMIs are compared to all within-sample UMIs corresponding to identical CDR3 amino acid sequence. Green: similar, but comparing to UMIs in another sample with identical CDR3. Red: pairwise comparison of randomly chosen UMI sequences from all samples. The similarity of all distributions indicates that the cleaning method removed erroneous sequences from our data. Note that there were only a limited number of CDR3s shared between samples, which may explain the visible difference to the other distributions.
