# Supplementary material for: Age and CMV-Infection Jointly Affect the EBV-Specific CD8+ T-Cell Repertoire
Source: Front Aging. 2021 Apr 29;2:665637. doi: 10.3389/fragi.2021.665637 (PMC9261403; doi:10.3389/fragi.2021.665637)
Supplement: Supplementary file 2 [file Data_Sheet_2.pdf]

Donor	Age	M/F	Number of UMIs	CDR3β	Vβ	Jβ
**1**	29	F	3	CASSPGGGGNEQFF	VB7-9	JB2-1
**3**	43	M	2	CASSPDLAGGPITSEQFF	VB4-3	JB2-1
**4**	51	M	3	CASSLAPGATNEKLFF	VB7-6	JB1-4
			14	CASSYSIPDGVFDEQFF	VB6-5/6	JB2-1
**5**	42	M	1	CASTRGGLTSSYNEQFF	VB12-3/4	JB2-1
**6**	29	F	2	CASSYATGTAYGYTF	VB6-5/6	JB1-2
**10**	30	F	2	CASSSANYGYTF	VB12-3	JB1-2
**11**	30	F	1	CAGSFGTADTGELFF	VB7-7	JB2-2
			1	CAISDPPVAGAYEQFF	VB10-3	JB2-1
			1	CAISDPPVAGAYEQYF	VB10-3	JB2-7
			2	CAISESGGWQFF	VB10-3	JB2-1
			1	CASSGGLAAPGTQYF	VB6-5/6	JB2-3
			1	CASSGTGTGELFF	VB6-1	JB2-2
			1	CASSHPLAANTGELFF	VB4-3	JB2-2
			1	CASSLAGANEQFF	VB7-9	JB2-1
			1	CASSLDDEQFF	VB12-3/4	JB2-1
			1	CASSLFRVQPQHF	VB6-3	JB1-5
			1	CASSPLSGANVLTF	VB7-6	JB2-6
			1	CASSPQVAGNEQFF	VB7-9	JB2-1
			1	CASSQDPTSNEQFF	VB4-2	JB2-1
			1	CASSYVQGINQPQHF	VB6-2	JB1-5
			1	CATAGRDGQPQHF	VB24-1	JB1-5
			1	CSAEGELANLDEQFF	VB20-1	JB2-1
			3	CSAPREGTSYGYTF	VB20-1	JB1-2
			1	CSARGGGSYNEQFF	VB20-1	JB2-1
			1	CSARGGRAADDYGFTL	VB20-1	JB1-2
			1	CSAWRAGALFV	VB20-1	JB2-2
			1	CSAWRSGELFF	VB20-1	JB2-2
			1	CSVDPTGGNTEAFF	VB29-1	JB1-1
			1	CSVGGLRTDTQYF	VB29-1	JB2-3
			1	CSVGPTGANQPQHF	VB29-1	JB1-5
			3	CSVVVVGNTGELFF	VB29-1	JB2-2
**14**	75	F	1	CAISESGGTDTQYF	VB10-3	JB2-3
			6	CASSLAPGTTNEKLFF	VB7-6	JB1-4
			1	CASSLHTTSGRAINTDTQYF	VB11-1	JB2-3
			1	CASSPLYADSHYGYTF	VB6-5/6	JB1-2
			1	CASSSANYGYTF	VB12-3/4	JB1-2
			92	CASSSVTEAFF	VB12-3/4	JB1-2
			1	CASSSVTEPFF	VB12-3/4	JB1-1
			1	CASSVLPQQGVDDQFF	VB4-1	JB2-1
			7	CASSVVNEQFF	VB12-3/4	JB2-1
			3	CASSYQTGTGVYGYTF	VB6-5/6	JB1-2
**17**	68	M	2	CASSLEVTGELFF	VB7-3	JB2-2
			1	CASSSVNEQFF	VB12-3/4	JB2-1
			1	CASSTVGGRDYGYTF	VB2	JB1-2
			1	CASSYATGASYGYTF	VB6-5/6	JB1-2
			1	CAWQSPGGDNEQFF	VB7-8	JB2-1
**18**	76	M	4	CSASTTSGAPSEQFF	VB20-1	JB2-1
**19**	67	M	1	CASISGHGYEEQYF	VB27	JB2-7
			194	CASISGQGYEEQYV	VB27	JB2-7
			1	CASSFRTVSSYEQYF	VB7-8	JB2-7
			1	CASSLAPEDLNKVLFF	VB7-6	JB1-4
			140	CASSLAPGATNEKLFF	VB7-6	JB1-4

			1	CASSPLGGAGGTDTQYF	VB9	JB2-3
			1	CASSQVWGSGEKLFF	VB3-1	JB1-4
			3	CASSTRDIGFYEQYF	VB7-9	JB2-7
			7	CSVEGSSAGRTLHEQYF	VB29-1	JB2-7
**20**	68	F	3	CASSALWPTNEKLFF	VB27	JB1-4
			7	CASSLAPGATNEKLFF	VB7-6	JB1-4
**21**	65	F	1	CASGLLLGGADTQYF	VB28	JB2-3
			1	CASSLLLGGADAQYF	VB28	JB2-3
			156	CASSLLLGGADTQYF	VB28	JB2-3
			2	CASSQTGFGNQPQHF	VB25-1	JB1-5
**22**	76	M	28	CASRVGTGTAREQFF	VB6-5/6	JB2-1
			10	CASSGGGTGAYEQYF	VB4-3	JB1-6
			116	CASSQEGNLGQGMGYSPLHF	VB4-3	JB1-6
			1	CASSQEGNLGQGMGYSPLRF	VB4-3	JB1-6
			1	CASSQEVNLGQGMGFSPLLF	VB4-3	JB1-6
			3	CASSSANYGYTF	VB12-3/4	JB1-2
			31	CASSVVGLAFF	VB12-3/4	JB1-1
			3	CAWVPGTGGFQAFF	VB30	JB1-1
**23**	81	M	3	CASSHTSGALYNEQFF	VB27	JB2-1
			3	CASSLAPGATNEKLFF	VB7-6	JB1-4
			11	CSARREFGSGSGLSYNEQFF	VB20-1	JB2-1

Supplementary Table 1: CMV^{A2-NLV}-specific TCR sequences

Donor	Age	M/F	Number of UMIs	CDR3β	Vβ	Jβ
**24**	41	F	1	CAIRGIEDRGVEAFF	VB10-3	JB1-1
			1	CASEGDTQYF	VB19	JB2-3
			1	CASRDRTGLQPQHF	VB6-5/6	JB1-5
			1	CASSDLPQSSGNTIYF	VB2	JB1-3
			1	CASSESTGVRLYEQFF	VB10-1	JB2-1
			1	CASSLQSLADSYEQYF	VB12-3/4	JB2-7
			2	CASSLVGGNIQYF	VB7-8	JB2-4
			1	CASSPGTGTYTEAFF	VB6-5/6	JB1-1
			1	CASSRRSRDQYEQYF	VB7-9	JB2-7
			1	CASSSRAGYNEQFF	VB14	JB2-1
			1	CASSVERWESNEQFF	VB9	JB2-1
			1	CATSRVVTGTETQYF	VB15	JB2-5
			1	CSARDLDGGELFF	VB20-1	JB2-2
			12	CSARDQTGNGYTF	VB20-1	JB1-2
			1	CSVAGLAGAPGFEQYF	VB29-1	JB2-7
			1	CSVGQGWNQPQHF	VB29-1	JB1-5
			2	CSVLRSTGELFF	VB29-1	JB2-2
**25**	42	F	1	CASSLGERTYAYEQYF	VB3-1	JB2-7
			1	CASSSDRLLAKNIQYF	VB2	JB2-4
			1	CASSVGSLTSGDEQFF	VB9	JB2-1
			5	CASSYGQQETQYF	VB12-3/4	JB2-5
			2	CSARAGTGNTIYF	VB20-1	JB1-3
			2	CSARDRMGNTIYF	VB20-1	JB1-3
			4	CSARDRTGNGYTF	VB20-1	JB1-2
			1	CSARDRVGNTIYF	VB20-1	JB1-3
			2	CSARDSIGNGYTF	VB20-1	JB1-2
			1	CSARDWIGNGYTF	VB20-1	JB1-2
			1	CSAREGTGNTIYF	VB20-	JB1-3
			1	CSARERTGNTIYF	VB20-1	JB1-3
			2	CSARSGTGNTIYF	VB20-1	JB1-3
			2	CSARVPPGNTIYF	VB20-1	JB1-3
			5	CSVGQGGTNEKLFF	VB29-1	JB1-4
			3	CSVGSGGTNEKLFF	VB29-1	JB1-4
			7	CSVGTGGTNEKLFF	VB29-1	JB1-4
**26**	49	M	1	CSAYRGRPETQYF	VB20-1	JB2-5
**27**	50	M	1	CAISELAGGSSYNEQFF	VB10-3	JB2-1
			1	CAISSYLSSSNQPQHF	VB10-3	JB1-5
			1	CASGLLQVQETQYF	VB12-5	JB2-5
			1	CASRAGSDEKLFF	VB7-9	JB1-4
			1	CASRPEPGQGVNEQYF	VB3-1	JB2-7
			1	CASRTQALSSYNSPLHF	VB27	JB1-6
			1	CASRTSGGSGELFF	VB7-9	JB2-2
			1	CASSAGGPEVAEQYF	VB10-2	JB2-7
			3	CASSDAQVLPSEQYF	VB2	JB2-7
			1	CASSEGGGANYGYTF	VB2	JB1-2
			1	CASSFGGGPYNEQFF	VB12-3/4	JB2-1
			1	CASSKDYEQYF	VB14	JB2-7
			1	CASSLDGATQYF	VB11-2	JB2-3
			1	CASSLGEGGDIQYF	VB4-	JB2-4
			2	CASSLGGPLGEQYF	VB7-8	JB2-7
			1	CASSLGGQGFYGYTF	VB7-9	JB1-2
			1	CASSLGGTGDHEQYF	VB12-3/4	JB2-7

			1	CASSLLSGNEQFF	VB5-5	JB2-1
			1	CASSLSGHYNEQFF	VB27	JB2-1
			1	CASSLSGVDTQYF	VB27	JB2-3
			1	CASSLSIPRSSYEQYF	VB27	JB2-7
			1	CASSLTSGGAGQYF	VB5-1	JB2-5
			1	CASSLVAGPNEQYF	VB7-9	JB2-7
			1	CASSLVALSGNTIYF	VB7-9	JB1-3
			1	CASSLVSGEYNEQFF	VB5-1	JB2-1
			1	CASSPDRVYGYTF	VB12-3/4	JB1-2
			2	CASSPDTNSPLHF	VB3-1	JB1-6
			1	CASSPGTGGFYEQYF	VB27	JB2-7
			1	CASSPPVPEAFF	VB7-9	JB1-1
			1	CASSPQGTEAFF	VB5-1	JB1-1
			1	CASSPRLAGGTDTQYF	VB5-1	JB2-3
			1	CASSPTGDDYGYTF	VB10-2	JB1-2
			1	CASSQKQGRSADTQYF	VB3-1	JB2-3
			1	CASSSMHSGANVLTF	VB19	JB2-6
			1	CASSSRPGGTTGELFF	VB9	JB2-2
			1	CASSYPTGQAPDTQYF	VB7-9	JB2-3
			1	CASSYSNTGHYGYTF	VB6-5/6	JB1-2
			1	CATSDSTGNQPQHF	VB24-1	JB1-5
			1	CATSTGTGGQPQHF	VB19	JB1-5
			1	CSARDPGTDYEQYF	VB20-1	JB2-7
			1	CSARHLWPDGGSGANVLTF	VB20-1	JB2-6
			1	CSARREGPITGELFF	VB20-1	JB2-2
			1	CSARRTDTEAFF	VB20-1	JB1-1
			1	CSASSSGELFF	VB20-1	JB2-2
			1	CSVEGTGDEQYF	VB29-1	JB2-7
			1	CSVGAGGTNEKLFF	VB29-1	JB1-4
			1	CSVGTGGTNEKLFF	VB29-1	JB1-4
**28**	24	F	2	CASTPRLAGGEEQYF	VB28	JB2-7
**29**	21	F	3	CSARDQTGNGYTF	VB20-1	JB1-2
**30**	24	F	1	CASNQIEGTEAFF	VB9	JB1-1
			3	CASSRPGSNQPQHF	VB7-9	JB1-5
			2	CASSSGQLTNTEAFF	VB9	JB1-1
**31**	28	F	2	CAISESVGRDSGYTF	VB10-3	JB1-2
			1	CASNSAGRAGETQYF	VB27	JB2-5
			1	CASSFGRANEQFF	VB12-3/4	JB2-1
			2	CASSFSGRVGTYNEQFF	VB11-2	JB2-1
			1	CASSGTTYEQYF	VB28	JB2-7
			1	CASSLDPGLPRGRGYTF	VB5-1	JB1-2
			2	CASSLDPQGVGNQPQHF	VB5-1	JB1-5
			1	CASSLITSYGYTF	VB12-3/4	JB1-2
			1	CASSLRDGEQFF	VB11-3	JB2-1
			1	CASSLSQGGEKLFF	VB27	JB1-4
			1	CASSPGPTRDYEQYV	VB5-1	JB2-7
			1	CASSPRNEETQYF	VB18	JB2-5
			1	CASSPSRGETQYF	VB7-2	JB2-5
			1	CASSPTQGARYGYTF	VB5-1	JB1-2
			1	CASSQEGLEETQYF	VB4-1	JB2-5
			1	CASSRPGMRGQNIQYF	VB7-2	JB2-4
			1	CASSSGTSGTEQYF	VB5-1	JB2-7
			1	CASSSRETYEQYF	VB11-1	JB2-7
			2	CASSVDPRGNEQFF	VB9	JB2-1
			1	CSAMRGTGRYEQYF	VB20-1	JB2-7
			1	CSAPPRSYSSSYNEQFF	VB20-1	JB2-1
			1	CSARDGRASGANVLTF	VB20-1	JB2-6

			2	CSARDRIGNGYTF	VB20-1	JB1-2
			1	CSARDRTGNGYTF	VB20-1	JB1-2
			1	CSARVTSIEAFF	VB20-1	JB1-1
			1	CSASPGPGREQFF	VB20-1	JB2-1
			1	CSASRQYTEAFF	VB20-1	JB1-1
			1	CSVGTGGTNEKLFF	VB29-1	JB1-4
			1	CSVGTGSTEAFF	VB29-1	JB1-1
**32**	41	M	2	CSARDGTGNGYTF	VB20-1	JB1-2
**33**	40	M	1	CAISYFLLAGDGTDTQYF	VB6-5/6	JB2-3
			1	CAKEGTNTEAFF	VB2	JB1-1
			1	CASGGDTNYGYTF	VB28	JB1-2
			1	CASIQETQYF	VB2	JB2-5
			1	CASLLRGENTEAFF	VB2	JB1-1
			1	CASRIGGTGDGEQYF	VB28	JB2-7
			1	CASRMGGTGDCELYL	VB28	JB2-7
			2	CASRPGQGIDEQYF	VB2	JB2-7
			1	CASRREGPDTQYF	VB7-9	JB2-3
			1	CASRRSLPEQFF	VB28	JB2-1
			1	CASSAGGHSNQPQHF	VB3-1	JB1-5
			1	CASSASGWRGYTF	VB6-5/6	JB1-2
			1	CASSCGGLNTEAFF	VB12-3/4	JB1-1
			1	CASSDLRTDLSNYGYTF	VB7-2	JB1-2
			1	CASSDRDRAPNQPQHF	VB2	JB1-5
			1	CASSDSRQGADTQYF	VB6-4	JB2-3
			1	CASSEDGMNTEAFF	VB10-2	JB1-1
			1	CASSESGQGWTEAFF	VB10-2	JB1-1
			10	CASSFGVSNTEAFF	VB12-3/4	JB1-1
			1	CASSFVNGGAGKFF	VB28	JB2-1
			1	CASSFVVRGQETQYF	VB5-1	JB2-5
			1	CASSHGAWNTEAFF	VB12-3/4	JB1-1
			1	CASSIGAGNSYEQYF	VB19	JB2-7
			2	CASSLDPTGGHHSSYNEQFF	VB11-2	JB2-1
			2	CASSLDSGGYNEQFF	VB7-6	JB2-1
			1	CASSLDVSRTWTGELFF	VB7-2	JB2-2
			1	CASSLEGPDNSPLHF	VB5-1	JB1-6
			1	CASSLGLAGSYNEQFF	VB5-1	JB2-1
			2	CASSLGLGTGELFF	VB11-2	JB2-2
			1	CASSLLTQGPSYEQYF	VB19	JB2-7
			1	CASSLSNNEQFF	VB5-6	JB2-1
			1	CASSPEASGANVLTF	VB6-2	JB2-6
			1	CASSPGRAYQETQYF	VB5-1	JB2-5
			2	CASSPHGRGSYSYEQYF	VB18	JB2-7
			1	CASSPQQGNTGELFF	VB19	JB2-2
			3	CASSPRAGWHYGYTF	VB18	JB1-2
			2	CASSPRTGVATNYGYTF	VB7-6	JB1-2
			1	CASSPTAESNQETQYF	VB11-2	JB2-5
			2	CASSPTLVTHYGYTF	VB6-5/6	JB1-2
			1	CASSPWGSGYNEKLFF	VB13	JB1-4
			1	CASSQELGGPRYF	VB4-2	JB2-3
			1	CASSQESGGTEYEQYF	VB3-1	JB2-7
			1	CASSQGFTDTQYF	VB3-1	JB2-3
			1	CASSQGLAGGSYNEQFF	VB3-1	JB2-1
			1	CASSQPRPGQGSFYGYTF	VB4-2	JB1-2
			1	CASSQSSYNEQFF	VB4-1	JB2-1
			4	CASSQTDIYNEQFF	VB4-1	JB2-1
			2	CASSSGLYEQYF	VB11-2	JB2-7
			1	CASSSGVSNTEAFF	VB12-3/4	JB1-1

			1	CASSSKQVNEQYF	VB7-9	JB2-7
			1	CASSSLRGVSRQYF	VB11-2	JB2-3
			2	CASSSPRLAGVVQETQYF	VB28	JB2-5
			1	CASSSTGQSTGELFF	VB12-3/4	JB2-2
			1	CASSYAGFNQPQHF	VB6-3	JB1-5
			4	CASTPGTLNTEAFF	VB12-3/4	JB1-1
			1	CATSDPTSGQETQYF	VB24-1	JB2-5
			1	CATSVDRDQDGETQYF	VB15	JB2-5
			1	CATSVSGGGDTQYF	VB15	JB2-3
			1	CAWSPGPIGTEAFF	VB30	JB1-1
			1	CSARDLAEGFKQYV	VB20-1	JB2-7
			1	CSARDVGGLSYNEQFF	VB20-1	JB2-1
			1	CSARENGDDTEELFF	VB20-1	JB2-2
			2	CSARFVSERYEQYF	VB20-1	JB2-7
			1	CSARPTGDPGNTIYF	VB20-1	JB1-3
			8	CSGTQGAEAFF	VB29-1	JB1-1
			1	CSVLAPLGTGNSTNTGELFF	VB29-1	JB2-2
			3	CSVNRGSGELFF	VB29-1	JB2-2
			1	CSWTQGAEAFF	VB29-1	JB1-1
**34**	52	M	1	CASSDTAVSPGELFF	VB2	JB2-2
			1	CSARDRGLGNTIYF	VB20-1	JB1-3
			1	CSASWGYTEAFF	VB20-1	JB1-1
**35**	70	M	1	CASSATLTGLNTEAFF	VB28	JB1-1
			1	CASSEAVTRGDTQYF	VB2	JB2-3
			1	CASSEVQGGSPLHF	VB6-1	JB1-6
			1	CASSLGYPGTNQPQHF	VB12-3/4	JB1-5
			1	CASSLSSGIAGEGGELFF	VB11-1	JB2-2
			1	CASSLTVGRDNEQFF	VB7-3	JB2-1
			3	CASSLWRDLAYNEQFF	VB11-1	JB2-1
			1	CASSPITLTGENYGYTF	VB6-5/6	JB1-2
			1	CASSPSPGGTQDTQYF	VB6-4	JB2-3
			1	CASSTPGTSLDTQYF	VB27	JB2-3
			1	CASSYSETQYF	VB6-5/6	JB2-5
**36**	71	F	2	CSARDQRTLFYEQYF	VB20-1	JB2-7
**37**	68	F	2	CASSVGGEAYEQYF	VB9	JB2-7
			1	CASTGDTLSYEQYF	VB6-3	JB2-7
**38**	64	M	1	CASSLAGTGSTDTQYF	VB7-2	JB2-3
			1	CASSLGTGNHEQFF	VB12-3/4	JB2-1
			2	CASSQSPGGTQYF	VB14	JB2-5
			1	CASSSPGTGTGANVLTF	VB7-8	JB2-6
**39**	74	M	1	CRVRTRGTHETLFF	VB29-1	JB1-4
			5	CSVGTGGTNEKLFF	VB29-1	JB1-4
**40**	79	F	2	CASSPGLVAPGEQYF	VB2	JB2-7
			1	CSGRTGAGGYTF	VB29-1	JB1-2
			2	CASSPGLVAPGEQYF	VB2	JB2-7
**41**	82		2	CASSPVGGPTNYGYTF	VB12-3/4	JB1-2
**42**	70	F	1	CASSAGQIAPGELFF	VB2	JB2-2
			2	CSARDGTGNGYTF	VB20-1	JB1-2
			1	CSARLIDPAGGHQETQYF	VB20-1	JB2-5
			4	CSVGSAGTNEKLFF	VB29-1	JB1-4
			1	CSVSGTGVMETQYF	VB29-1	JB2-5
**43**	60	F	1	CASGDEGASGYTF	VB9	JB1-2
			1	CASSFRGPGNTIYF	VB28	JB1-3
			1	CASSHGSSYNEQFF	VB7-9	JB2-1
			1	CASSLLGEGDTQYF	VB13	JB2-3
			1	CASSLSGGGEASYEQYF	VB4-1	JB2-7

1	CASSLSSLSSYNEQFF	VB13	JB2-1
1	CASSQDRGITEAFF	VB4-1	JB1-1
1	CASSSGGEDYEQYF	VB12-3/4	JB2-7
3	CASSSLDTSPHEQYF	VB11-2	JB2-7
1	CSAPIADGAYEQYF	VB20-1	JB2-7
1	CSARDSGDPGYTF	VB20-1	JB1-2
1	CSASDAGGPGYAF	VB20-1	JB1-2
1	CSASHVQGFSYGYTF	VB20-1	JB1-2
2	CSPRPGRGVTEAFF	VB20-1	JB1-1

Supplementary Table 2A: EBV^{A2-GLC}-specific TCR sequences of CMV- individuals

Donor	Age	M/F	Number of UMIs	CDR3β	Vβ	Jβ
**1**	29	F	1	CAIRSGYSNQPQHF	VB28	JB1-5
			1	CASGRGYEQYF	VB5-1	JB2-7
			1	CASNQLGGGIDTQYF	VB7-2	JB2-3
			2	CASSFSGDPSYEQYF	VB12-3/4	JB2-7
			1	CASSLQVGSSYNEQFF	VB7-6	JB2-1
			1	CASSPALHPSSYNEQFF	VB7-2	JB2-1
			1	CASSPTAGPGSYEQYF	VB7-6	JB2-7
			1	CASSSTRAGGRDYGYTF	VB28	JB1-2
			1	CSARDASTGEQETQYF	VB20-1	JB2-5
			1	CSARKRSSYNEQFF	VB20-1	JB2-1
			1	CSASLGNEQFF	VB20-1	JB2-1
**2**	49	F	1	CASSELDPGSTEAFF	VB5-1	JB1-1
			1	CASSFSSGDTEAFF	VB28	JB1-1
			1	CASSLAVEQFF	VB7-9	JB2-1
			1	CASSLEGQGGTEAFF	VB13	JB1-1
			1	CASSLRGPRNSPLHF	VB7-6	JB1-6
			1	CASSQDTRLAVTGELFF	VB4-3	JB2-2
			1	CASSRDRGTGELFF	VB5-5	JB2-2
			1	CATNDRDGPTDTQYF	VB24-1	JB2-3
			1	CATSDLTGDEQFF	VB24-1	JB2-1
			5	CSARIGVGNTIYF	VB20-1	JB1-3
			5	CSARVGVGNTIYF	VB20-1	JB1-3
**4**	51	M	1	CASSLARTQETQYF	VB7-9	JB2-5
			1	CASSLSRGLLNGYTF	VB27	JB1-2
			1	CSVAGDRAEAFF	VB29-1	JB1-1
**5**	42	M	1	CASNQIEGTEAFF	VB9	JB1-1
			3	CASSRPGSNQPQHF	VB7-9	JB1-5
			2	CASSSGQLTNTEAFF	VB9	JB1-1
**6**	29	F	1	CASSPTSGSIYEQYF	VB3-1	JB2-7
			1	CASSVEGATGSSYEQYF	VB9	JB2-7
			3	CAWRDDGTDTQYF	VB7-8	JB2-3
			2	CSARDRGLGNTIYF	VB20-1	JB1-3
			2	CSARDRGMGNTIYF	VB20-1	JB1-3
			3	CSARDRGVGNTIYF	VB20-1	JB1-3
			1	CSVEAGGRVYNEQFF	VB29-1	JB2-1
			2	CSVGAGPTNEKLFF	VB29-1	JB1-4
**7**	27	F	2	CAISGTGRVDEQFF	VB10-3	JB2-1
			1	CASSQITGDNQPQHF	VB4-1	JB1-5
			2	CASSRTGNNSPLHF	VB11-1	JB1-6
			1	CASSVGSVGATYEQYF	VB7-6	JB2-7
			1	CSARDRALSVYEQYF	VB20-1	JB2-7
**8**	52	F	1	CAIGTSPSTDTQYF	VB10-3	JB2-3
			1	CASREPYRGSNGGQETQYF	VB2	JB2-5
			3	CASRVPGLAGAQPELFF	VB5-1	JB2-2
			1	CASSDGRATGELFF	VB6-1	JB2-2
			1	CASSDWDKTGFYEQYF	VB6-1	JB2-7
			1	CASSERQGTRADEQYF	VB6-1	JB2-7
			1	CASSLEGGTLQETQYF	VB5-6	JB2-5
			1	CASSLGLHSIQGYEQYF	VB7-9	JB2-7
			1	CASSLQGGNYGYTF	VB27	JB1-2
			1	CASSPEELFSYEQYF	VB9	JB2-7
			1	CASSPQDRAGGDEQFF	VB4-1	JB2-1

			1	CASSPYGGDTQYF	VB12-3/4	JB2-3
			1	CASSSPVRDTQYF	VB7-9	JB2-3
			1	CASSWTGGREQFF	VB7-8	JB2-1
			4	CATSRVGGFNEQFF	VB15	JB2-1
**9**	45	M	1	CSARDTTGNGYTF	VB20-1	JB1-2
**10**	30	F	1	CSVGSGGTNEKLFF	VB29-1	JB1-4
**11**	30	F	1	CASSLAIGEQFF	VB5-4	JB2-1
**12**	48	F	1	CSARDRGLGNTIYF	VB20-1	JB1-3
**13**	25	F	1	CSVGTGGTNEKLFF	VB29-1	JB1-4
**14**	74	F	1	CASSLGVGNSNQPQHF	VB7-8	JB1-5
			1	CSASQGAQEAFF	VB20-1	JB1-1
			1	CSVGTGGTNEKLFF	VB29-1	JB1-4
**15**	67	F	2	CAGSLAGRTEAFF	VB7-9	JB1-1
			1	CASGVANEQYF	VB7-8	JB2-7
			2	CASSIDGTAYEQYF	VB19	JB2-7
			1	CASSQGGLWGTEAFF	VB4-3	JB1-1
			1	CATSRIAGETQYF	VB15	JB2-5
			1	CAWTVNEKLFF	VB30	JB1-4
			1	CAWTVNEKLLF	VB30	JB1-4
			1	CSVDSGNYNEQFF	VB29-1	JB2-1
**16**	70	F	2	CAWSFSDIMNTEAFF	VB30	JB1-1
**17**	68	F	2	CSAATARIETQYF	VB29-1	JB2-5
**19**	67	M	1	CASSFYEGQGATEQFF	VB11-2	JB2-1
			6	CASSLGAGSNYGYTF	VB27	JB1-2
			1	CASSPPSATYEQYF	VB18	JB2-7
			1	CASSQEAEREQFF	VB4-3	JB2-1
			12	CASSTRDIGFYEQYF	VB7-9	JB2-7
			1	CSAPGQGLMNTEAFF	VB20-1	JB1-1
**20**	68	F	4	CASSAGQVAPGELFF	VB2	JB2-2
			3	CSARDRTGNGYTF	VB20-1	JB1-2
			1	CSAYRGRPETQYF	VB20-1	JB2-5
			3	CSVGTGGTNEKLFF	VB29-1	JB1-4
**21**	65	F	2	CASSAVLAGEETQYF	VB12-3/4	JB2-5
			1	CASSEGRVAPGEQYF	VB2	JB2-7
			1	CASSEWTGYQPQHF	VB6-1	JB1-5
			1	CASSGTTTNEKLFF	VB12-3/4	JB1-4
			1	CASSLLLGGADTQYF	VB28	JB2-3
			2	CASSQDYRAGDEQYF	VB4-3	JB2-7
			1	CASSSPTGQANYGYTF	VB7-9	JB1-2
			1	CASSYRTAANEKLFF	VB6-5/6	JB1-4
			2	CASSYSGTVGYTF	VB27	JB1-2
			1	CAWSSRGRAQETQYF	VB30	JB2-5
			1	CSASRSEGSNQPQHF	VB20-1	JB1-5
			13	CSVGAGGTNEKLFF	VB29-1	JB1-4
			2	CSVGTGGTNEKLFF	VB29-1	JB1-4
			15	CSVRGEGDTQYF	VB29-1	JB2-3

Supplementary Table 2B: EBV$^{A2\text{-}GLC}$-specific TCR sequences of CMV+ individuals
