# Supplementary material for: Age and CMV-Infection Jointly Affect the EBV-Specific CD8+ T-Cell Repertoire
Source: Front Aging. 2021 Apr 29;2:665637. doi: 10.3389/fragi.2021.665637 (PMC9261403; doi:10.3389/fragi.2021.665637)
Supplement: Supplementary file 3 [file Image_1.pdf]

Supplementary Figure 1: Flowchart donors

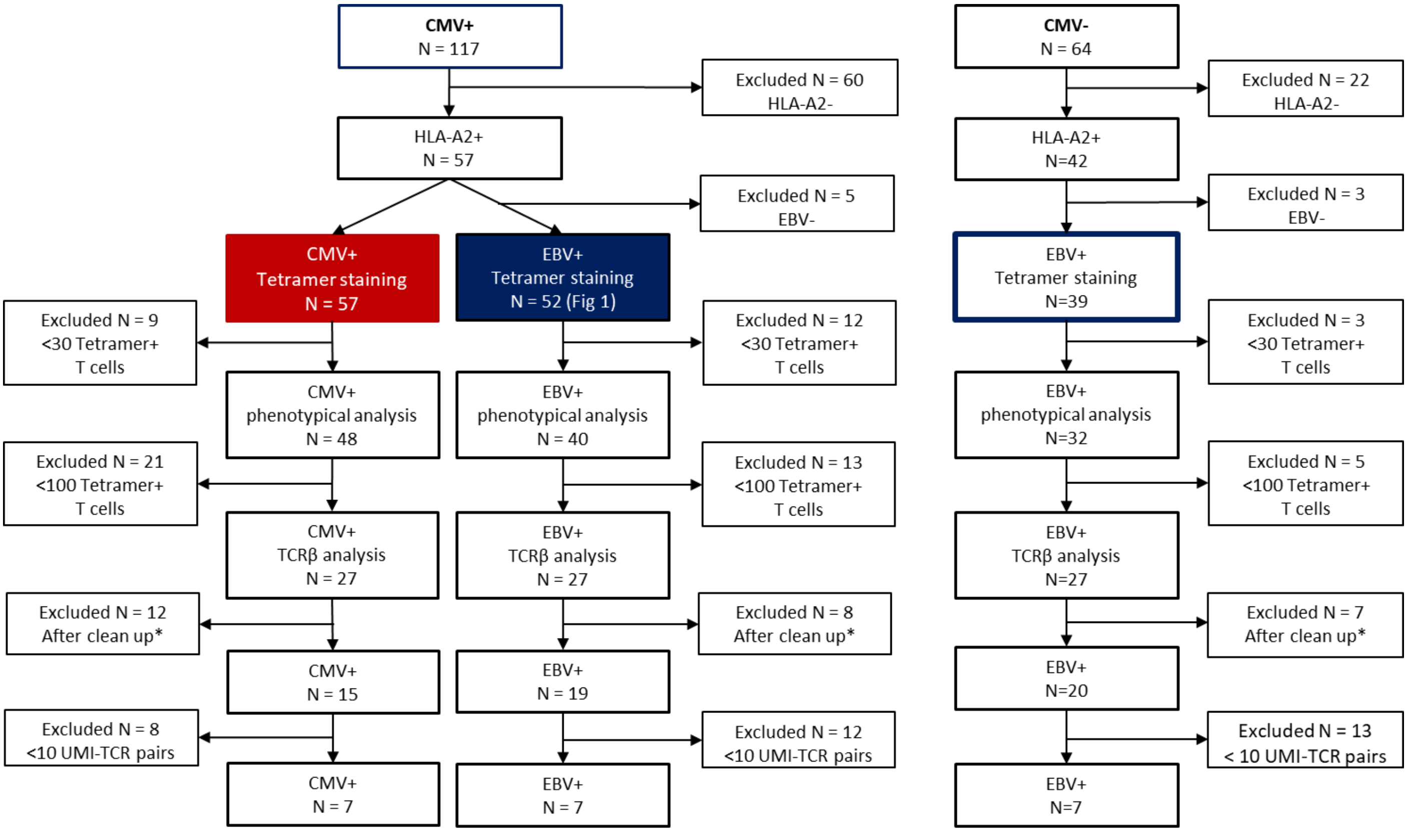

Supplementary Figure 1: Participants Flowchart
Overview of the participants and criteria used for the study. For the CMV+ individuals 54 participants (age 18-52) came from the study of Rosendahl Huber et al, whereas 63 participants (age 60 years and older) were derived from Kaaijk et al. For the CMV- individuals this was 28 and 36 respectively.
*Data shown in Supplementary figure 2.

Supplementary Figure 2

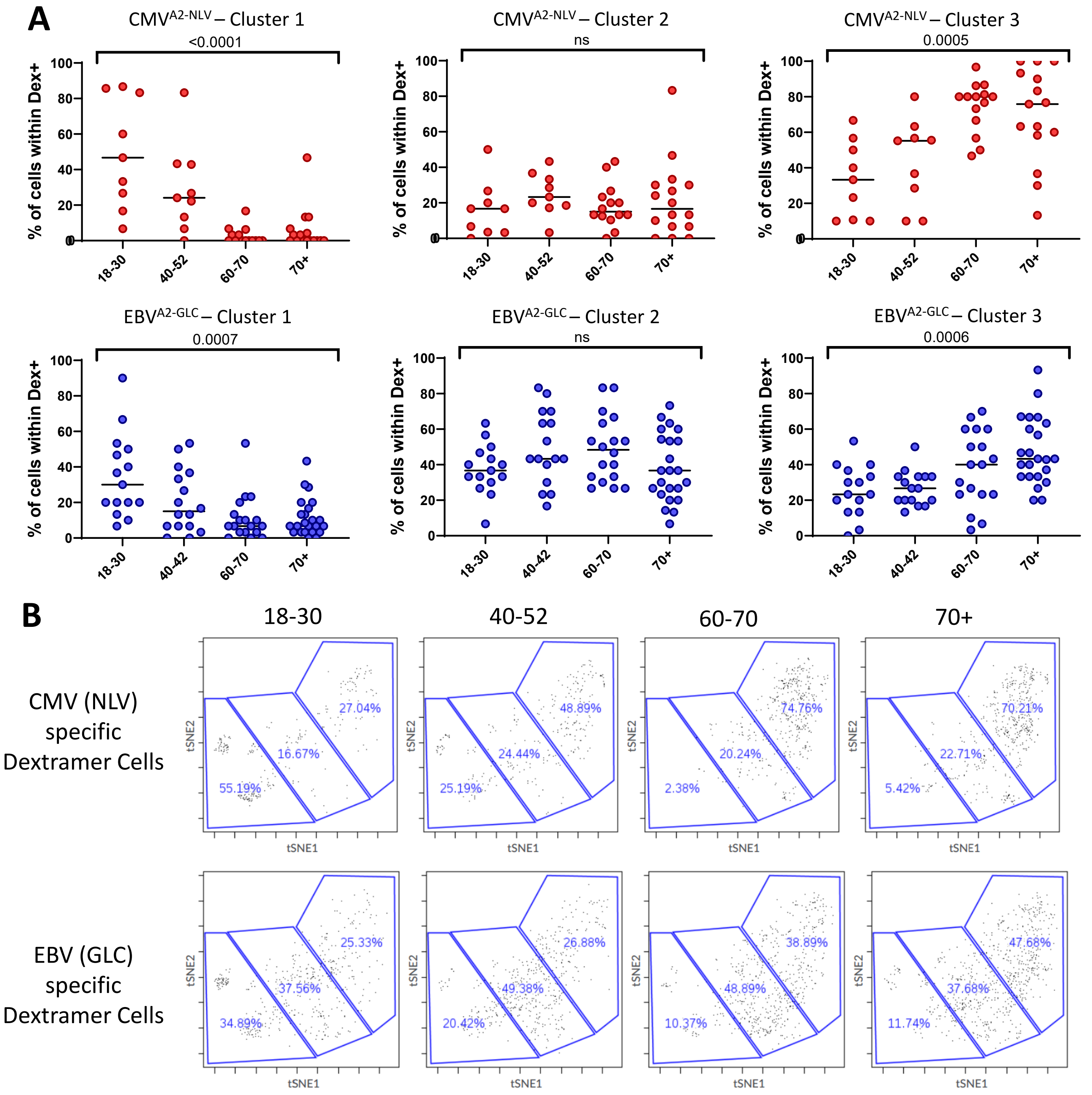

Supplementary Figure 2: Additional information associated with the t-SNE cluster analyses
A) Percentages of CD8+ T cells per t-SNE cluster (1, 2 and 3) for the different age groups of both CMVA2-NLV-specific (red, upper panels) and EBVA2-GLC-specific (blue, lower panels) CD8+ T cells. Horizontal lines show group medians. Differences between groups was tested with the Kruskal-Wallis test.
B) t-SNE analysis of CMVA2-NLV-specific (n=48) and EBVA2-GLC-specific (n=72) CD8+ T cells of donors categorized in 4 age groups, shown as dots. Clustering is based on MFI of CD5, PD-1, CD57, KLRG-1, CXCR3, CCR7, CD45RO, CD95, CD27 and CD127. Equal numbers of cells were used per sample

Supplementary Figure 3

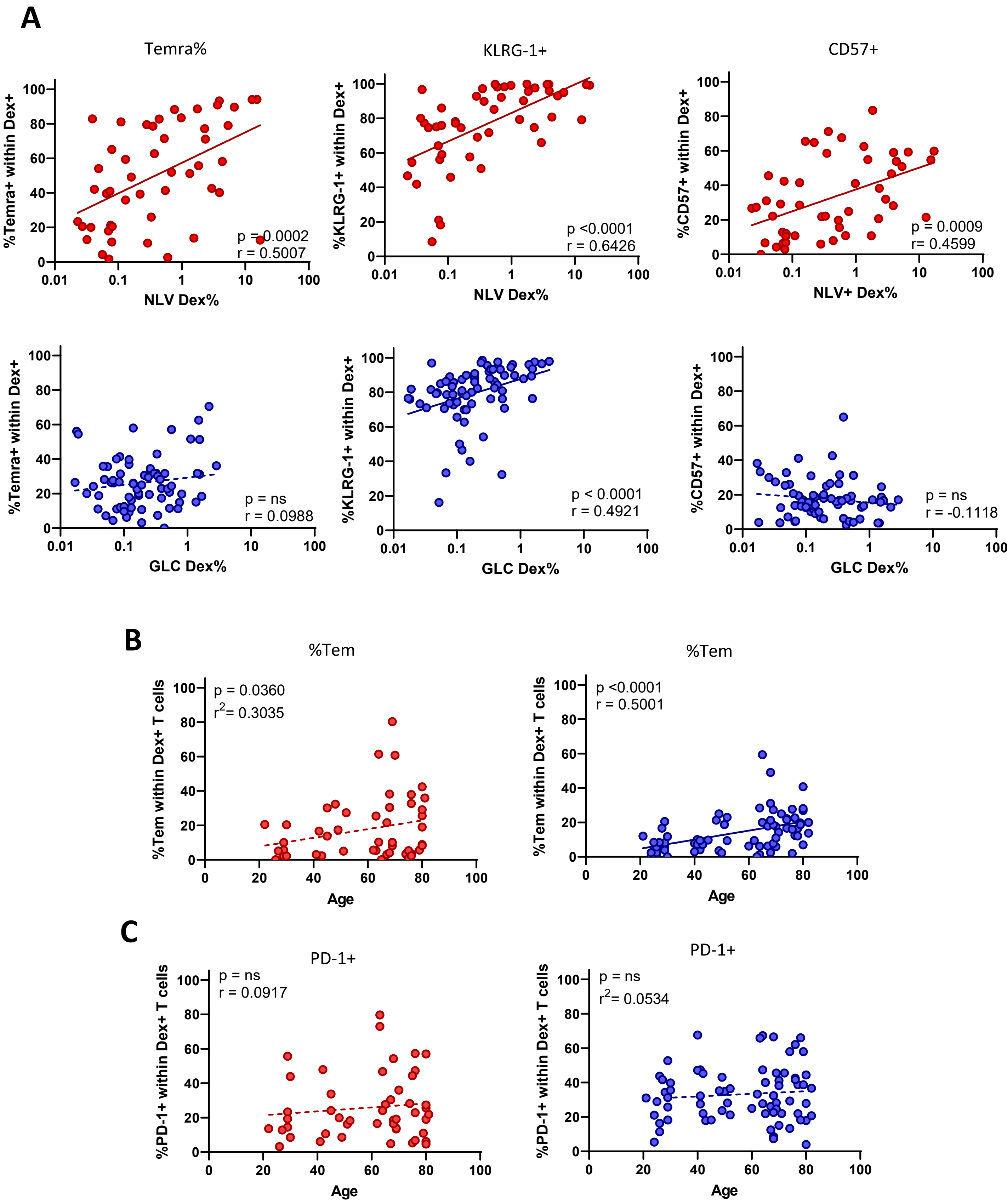

Supplementary Figure 3: Expression of the a more differentiated phenotype is associated with higher expression of the frequencies of $CMV^{A2\text{-}NLV}$ –specific and $EBV^{A2\text{-}GLC}$-specific T cells.
A) Correlation plots of percentage of Temra cells (left panel) CD57+ (middle panel) and KLRG-1+ (right panel) of CMVA2-NLV-specific (red, upper panels) and EBVA2-GLC-specific (blue, lower panels) CD8+ T cells and the frequencies of Dextramer+ T cells. Frequencies were log transformed.
Correlations were tested with Spearman's rank correlation .
Only donors with a sufficient T-cell response (at least 25 cells) were used for the phenotypical staining.
B+C) Percentage of Effector memory T cells (Tem, CD27-CD45RO+) (A) and PD-1+ T cells (B) of CMVA2-NLV-specific (red, left panel) and EBVA2-GLC-specific (blue, right panel) CD8+ T cells plotted with age. Solid lines indicate a slope significantly ($p<0.05$) different from a slope of 0, whereas a dotted line indicates no significant difference.

Supplementary Figure 4: Characterization of the antigen-specific TCRβ repertoire consisting of less than 10 UMI-TCR pairs.
A) T-cell repertoire of CMVA2-NLV-specific CD8+ T cells (n=8).
B+C) T-cell repertoire of EBVA2-GLC-specific CD8+ T cells of both CMV- (B) and CMV+ individuals (C). Each pie depicts the repertoire of a different donor. Colors represent shared CDR3 sequences between donors. Grey scales depict unique TCRβ sequences.

Supplementary Figure 4

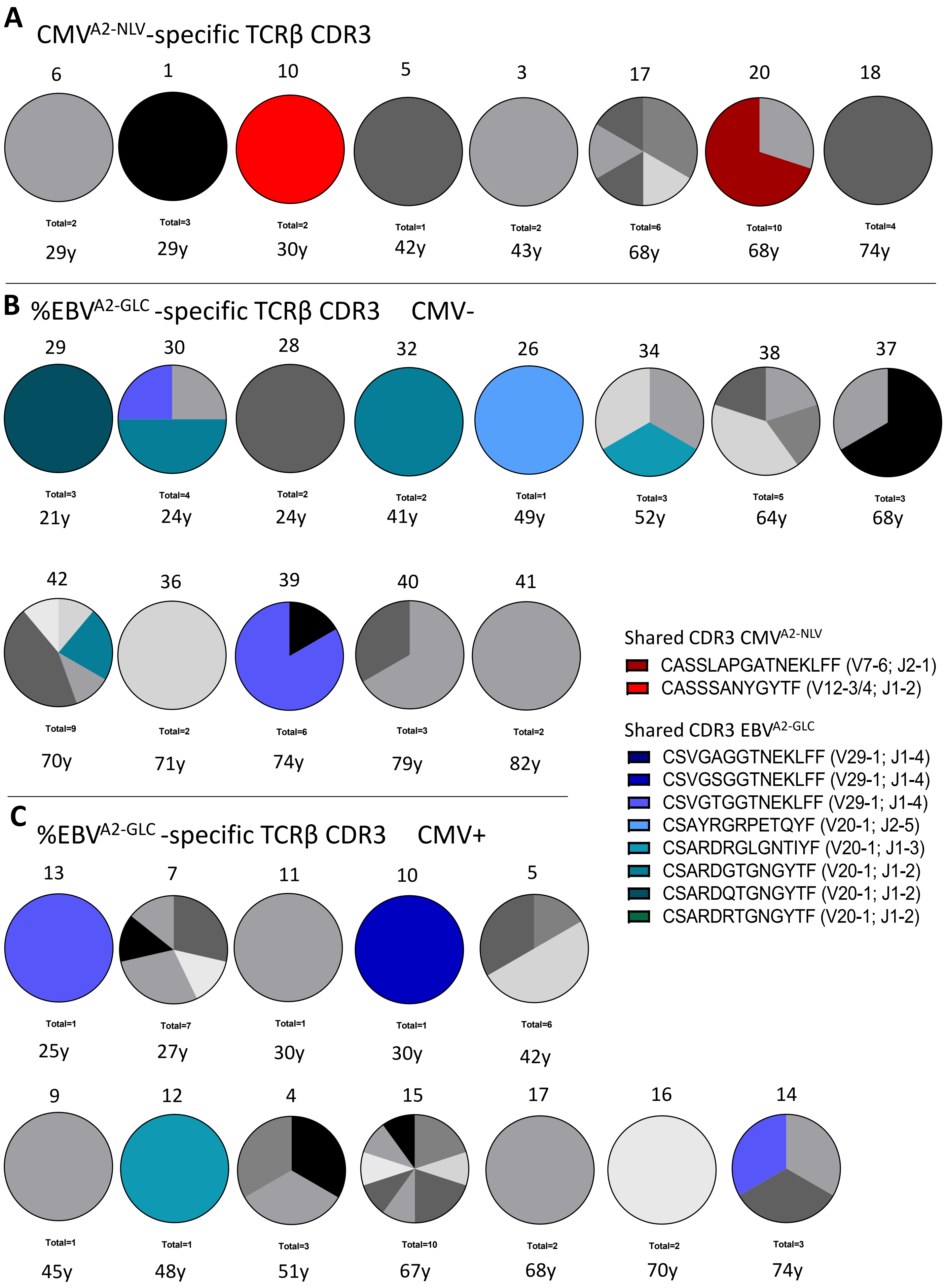

Supplementary Figure 5

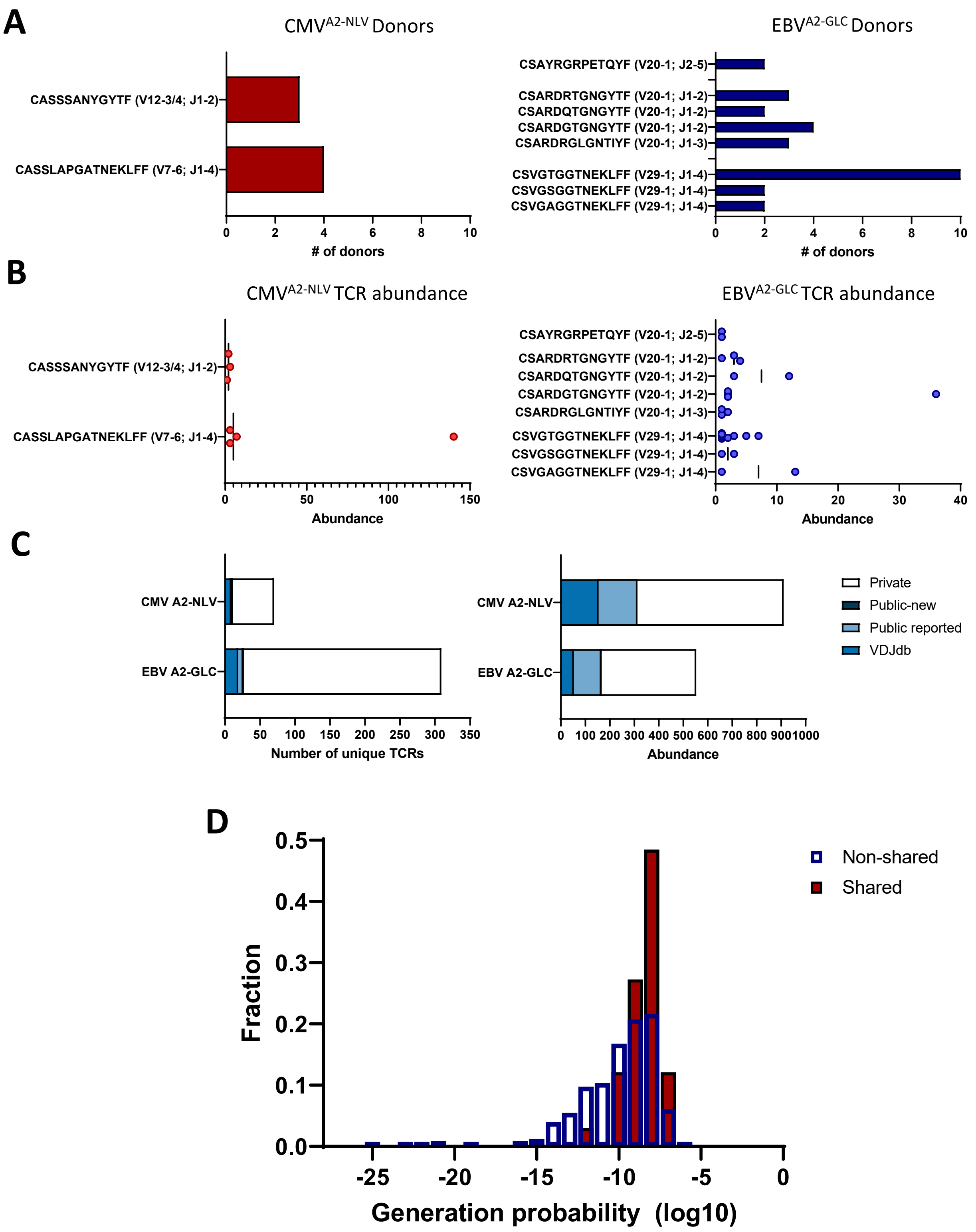

Supplementary Figure 5: Shared sequences consist of a higher generation probability.

A) Number of donors sharing TCRβ sequences in their CMV$^{A2\text{-}NLV}$-specific (red, upper panel) or EBV$^{A2\text{-}GLC}$-specific (blue, lower panel) T-cell repertoire.

B) Total number of different UMIs coupled to the shared TCRβ sequences found in the different donors of in their CMV$^{A2\text{-}NLV}$-specific (red, upper panel) or EBV$^{A2\text{-}GLC}$-specific (blue, lower panel) T-cell repertoire.

C) Number of total TCRβ sequences that are private (white) or shared within our data base (dark blue), and also reported in the VDJdb (light blue) or sequences not shared in our database but reported in the VDJdb (blue).

D) Generation probability distributions of sequences that are shared between donors within our data, or with the VDJdb (shared) and the other sequences (non-shared). Only sequences matching with the CMV$^{A2\text{-}NLV}$ and EBV$^{A2\text{-}GLC}$ epitopes were labeled as shared.

Supplementary Figure 6

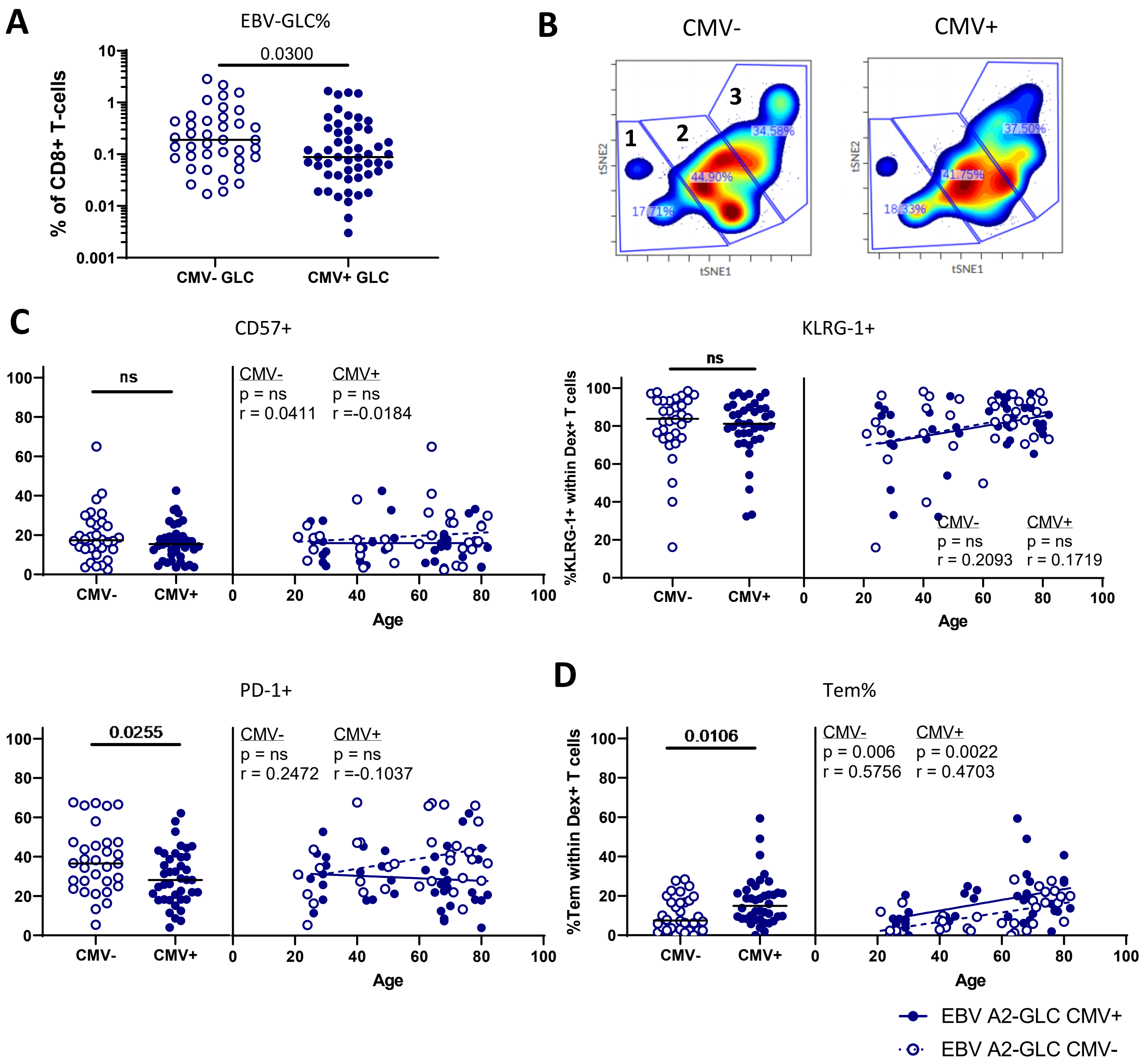

Supplementary Figure 6: Phenotypical differences between the EBV$^{A2\text{-}GLC}$-specific T cells in CMV^{-} and CMV^{+} individuals.

A) Percentage of EBV$^{A2\text{-}GLC}$-specific CD8^{+} T cells In CMV^{-} (blue, open circles) and CMV^{+} (blue, filled circles) individuals. Median is presented in the figures. Difference was compared by Mann Whitney U test.

B) t-SNE analysis of EBV$^{A2\text{-}GLC}$-specific CD8^{+} T cells based on MFI of CD5, PD-1, CD57, KLRG-1, CXCR3, CCR7, CD45RO, CD95, CD27 and CD127 in CMV- (n=32) and CMV^{+} individuals (n = 40). 30 cells were used from each sample. 3 large clusters were identified.

C) Percentage of CD57^{+} (upper left panel) KLRG-1+ (upper right panel) and PD-1^{+} (lower left panel) and fraction of Effector Memory T cells **(D)** of EBV$^{A2\text{-}GLC}$-specific CD8^{+} T cells shown for CMV^{-} (blue, open circles) and CMV^{+} (blue, filled circles) individuals (left panels) and correlated against the age of the donors (right panels). Median is presented in the figures. Differences were compared by Mann Whitney U test.
